# Development and validation of HERWIG 7 tunes from CMS underlying-event measurements

**DOI:** 10.1140/epjc/s10052-021-08949-5

**Published:** 2021-04-12

**Authors:** A. M. Sirunyan, A. Tumasyan, W. Adam, F. Ambrogi, T. Bergauer, M. Dragicevic, J. Erö, A. Escalante Del Valle, R. Frühwirth, M. Jeitler, N. Krammer, L. Lechner, D. Liko, T. Madlener, I. Mikulec, F. M. Pitters, N. Rad, J. Schieck, R. Schöfbeck, M. Spanring, S. Templ, W. Waltenberger, C.-E. Wulz, M. Zarucki, V. Chekhovsky, A. Litomin, V. Makarenko, J. Suarez Gonzalez, M. R. Darwish, E. A. De Wolf, D. Di Croce, X. Janssen, T. Kello, A. Lelek, M. Pieters, H. Rejeb Sfar, H. Van Haevermaet, P. Van Mechelen, S. Van Putte, N. Van Remortel, F. Blekman, E. S. Bols, S. S. Chhibra, J. D’Hondt, J. De Clercq, D. Lontkovskyi, S. Lowette, I. Marchesini, S. Moortgat, A. Morton, Q. Python, S. Tavernier, W. Van Doninck, P. Van Mulders, D. Beghin, B. Bilin, B. Clerbaux, G. De Lentdecker, H. Delannoy, B. Dorney, L. Favart, A. Grebenyuk, A. K. Kalsi, I. Makarenko, L. Moureaux, L. Pétré, A. Popov, N. Postiau, E. Starling, L. Thomas, C. Vander Velde, P. Vanlaer, D. Vannerom, L. Wezenbeek, T. Cornelis, D. Dobur, M. Gruchala, I. Khvastunov, M. Niedziela, C. Roskas, K. Skovpen, M. Tytgat, W. Verbeke, B. Vermassen, M. Vit, G. Bruno, F. Bury, C. Caputo, P. David, C. Delaere, M. Delcourt, I. S. Donertas, A. Giammanco, V. Lemaitre, K. Mondal, J. Prisciandaro, A. Taliercio, M. Teklishyn, P. Vischia, S. Wuyckens, J. Zobec, G. A. Alves, G. Correia Silva, C. Hensel, A. Moraes, W. L. Aldá Júnior, E. Belchior Batista Das Chagas, H. Brandao Malbouisson, W. Carvalho, J. Chinellato, E. Coelho, E. M. Da Costa, G. G. Da Silveira, D. De Jesus Damiao, S. Fonseca De Souza, J. Martins, D. Matos Figueiredo, M. Medina Jaime, M. Melo De Almeida, C. Mora Herrera, L. Mundim, H. Nogima, P. Rebello Teles, L. J. Sanchez Rosas, A. Santoro, S. M. Silva Do Amaral, A. Sznajder, M. Thiel, E. J. Tonelli Manganote, F. Torres Da Silva De Araujo, A. Vilela Pereira, C. A. Bernardes, L. Calligaris, T. R. Fernandez Perez Tomei, E. M. Gregores, D. S. Lemos, P. G. Mercadante, S. F. Novaes, S. S. Padula, A. Aleksandrov, G. Antchev, I. Atanasov, R. Hadjiiska, P. Iaydjiev, M. Misheva, M. Rodozov, M. Shopova, G. Sultanov, M. Bonchev, A. Dimitrov, T. Ivanov, L. Litov, B. Pavlov, P. Petkov, A. Petrov, W. Fang, Q. Guo, H. Wang, L. Yuan, M. Ahmad, Z. Hu, Y. Wang, E. Chapon, G. M. Chen, H. S. Chen, M. Chen, D. Leggat, H. Liao, Z. Liu, R. Sharma, A. Spiezia, J. Tao, J. Thomas-wilsker, J. Wang, H. Zhang, S. Zhang, J. Zhao, A. Agapitos, Y. Ban, C. Chen, A. Levin, Q. Li, M. Lu, X. Lyu, Y. Mao, S. J. Qian, D. Wang, Q. Wang, J. Xiao, Z. You, X. Gao, M. Xiao, C. Avila, A. Cabrera, C. Florez, J. Fraga, A. Sarkar, M. A. Segura Delgado, J. Jaramillo, J. Mejia Guisao, F. Ramirez, J. D. Ruiz Alvarez, C. A. Salazar González, N. Vanegas Arbelaez, D. Giljanovic, N. Godinovic, D. Lelas, I. Puljak, T. Sculac, Z. Antunovic, M. Kovac, V. Brigljevic, D. Ferencek, D. Majumder, B. Mesic, M. Roguljic, A. Starodumov, T. Susa, M. W. Ather, A. Attikis, E. Erodotou, A. Ioannou, G. Kole, M. Kolosova, S. Konstantinou, G. Mavromanolakis, J. Mousa, C. Nicolaou, F. Ptochos, P. A. Razis, H. Rykaczewski, H. Saka, D. Tsiakkouri, M. Finger, M. Finger Jr., A. Kveton, J. Tomsa, E. Ayala, E. Carrera Jarrin, H. Abdalla, Y. Assran, A. Mohamed, M. A. Mahmoud, Y. Mohammed, S. Bhowmik, A. Carvalho Antunes De Oliveira, R. K. Dewanjee, K. Ehataht, M. Kadastik, M. Raidal, C. Veelken, P. Eerola, L. Forthomme, H. Kirschenmann, K. Osterberg, M. Voutilainen, E. Brücken, F. Garcia, J. Havukainen, V. Karimäki, M. S. Kim, R. Kinnunen, T. Lampén, K. Lassila-Perini, S. Laurila, S. Lehti, T. Lindén, H. Siikonen, E. Tuominen, J. Tuominiemi, P. Luukka, T. Tuuva, C. Amendola, M. Besancon, F. Couderc, M. Dejardin, D. Denegri, J. L. Faure, F. Ferri, S. Ganjour, A. Givernaud, P. Gras, G. Hamel de Monchenault, P. Jarry, B. Lenzi, E. Locci, J. Malcles, J. Rander, A. Rosowsky, M. Ö. Sahin, A. Savoy-Navarro, M. Titov, G. B. Yu, S. Ahuja, F. Beaudette, M. Bonanomi, A. Buchot Perraguin, P. Busson, C. Charlot, O. Davignon, B. Diab, G. Falmagne, R. Granier de Cassagnac, A. Hakimi, I. Kucher, A. Lobanov, C. Martin Perez, M. Nguyen, C. Ochando, P. Paganini, J. Rembser, R. Salerno, J. B. Sauvan, Y. Sirois, A. Zabi, A. Zghiche, J.-L. Agram, J. Andrea, D. Bloch, G. Bourgatte, J.-M. Brom, E. C. Chabert, C. Collard, J.-C. Fontaine, D. Gelé, U. Goerlach, C. Grimault, A.-C. Le Bihan, P. Van Hove, E. Asilar, S. Beauceron, C. Bernet, G. Boudoul, C. Camen, A. Carle, N. Chanon, D. Contardo, P. Depasse, H. El Mamouni, J. Fay, S. Gascon, M. Gouzevitch, B. Ille, Sa. Jain, I. B. Laktineh, H. Lattaud, A. Lesauvage, M. Lethuillier, L. Mirabito, L. Torterotot, G. Touquet, M. Vander Donckt, S. Viret, T. Toriashvili, Z. Tsamalaidze, L. Feld, K. Klein, M. Lipinski, D. Meuser, A. Pauls, M. Preuten, M. P. Rauch, J. Schulz, M. Teroerde, D. Eliseev, M. Erdmann, P. Fackeldey, B. Fischer, S. Ghosh, T. Hebbeker, K. Hoepfner, H. Keller, L. Mastrolorenzo, M. Merschmeyer, A. Meyer, P. Millet, G. Mocellin, S. Mondal, S. Mukherjee, D. Noll, A. Novak, T. Pook, A. Pozdnyakov, T. Quast, M. Radziej, Y. Rath, H. Reithler, J. Roemer, A. Schmidt, S. C. Schuler, A. Sharma, S. Wiedenbeck, S. Zaleski, C. Dziwok, G. Flügge, W. Haj Ahmad, O. Hlushchenko, T. Kress, A. Nowack, C. Pistone, O. Pooth, D. Roy, H. Sert, A. Stahl, T. Ziemons, H. Aarup Petersen, M. Aldaya Martin, P. Asmuss, I. Babounikau, S. Baxter, O. Behnke, A. Bermúdez Martínez, A. A. Bin Anuar, K. Borras, V. Botta, D. Brunner, A. Campbell, A. Cardini, P. Connor, S. Consuegra Rodríguez, V. Danilov, A. De Wit, M. M. Defranchis, L. Didukh, D. Domínguez Damiani, G. Eckerlin, D. Eckstein, T. Eichhorn, A. Elwood, L. I. Estevez Banos, E. Gallo, A. Geiser, A. Giraldi, A. Grohsjean, M. Guthoff, A. Harb, A. Jafari, N. Z. Jomhari, H. Jung, A. Kasem, M. Kasemann, H. Kaveh, C. Kleinwort, J. Knolle, D. Krücker, W. Lange, T. Lenz, J. Lidrych, K. Lipka, W. Lohmann, R. Mankel, I.-A. Melzer-Pellmann, J. Metwally, A. B. Meyer, M. Meyer, M. Missiroli, J. Mnich, A. Mussgiller, V. Myronenko, Y. Otarid, D. Pérez Adán, S. K. Pflitsch, D. Pitzl, A. Raspereza, A. Saggio, A. Saibel, M. Savitskyi, V. Scheurer, P. Schütze, C. Schwanenberger, R. Shevchenko, A. Singh, R. E. Sosa Ricardo, H. Tholen, N. Tonon, O. Turkot, A. Vagnerini, M. Van De Klundert, R. Walsh, D. Walter, Y. Wen, K. Wichmann, C. Wissing, S. Wuchterl, O. Zenaiev, R. Zlebcik, R. Aggleton, S. Bein, L. Benato, A. Benecke, K. De Leo, T. Dreyer, A. Ebrahimi, M. Eich, F. Feindt, A. Fröhlich, C. Garbers, E. Garutti, P. Gunnellini, J. Haller, A. Hinzmann, A. Karavdina, G. Kasieczka, R. Klanner, R. Kogler, V. Kutzner, J. Lange, T. Lange, A. Malara, J. Multhaup, C. E. N. Niemeyer, A. Nigamova, K. J. Pena Rodriguez, O. Rieger, P. Schleper, S. Schumann, J. Schwandt, D. Schwarz, J. Sonneveld, H. Stadie, G. Steinbrück, B. Vormwald, I. Zoi, M. Baselga, S. Baur, J. Bechtel, T. Berger, E. Butz, R. Caspart, T. Chwalek, W. De Boer, A. Dierlamm, A. Droll, K. El Morabit, N. Faltermann, K. Flöh, M. Giffels, A. Gottmann, F. Hartmann, C. Heidecker, U. Husemann, M. A. Iqbal, I. Katkov, P. Keicher, R. Koppenhöfer, S. Maier, M. Metzler, S. Mitra, M. U. Mozer, D. Müller, Th. Müller, M. Musich, G. Quast, K. Rabbertz, J. Rauser, D. Savoiu, D. Schäfer, M. Schnepf, M. Schröder, D. Seith, I. Shvetsov, H. J. Simonis, R. Ulrich, M. Wassmer, M. Weber, C. Wöhrmann, R. Wolf, S. Wozniewski, G. Anagnostou, P. Asenov, G. Daskalakis, T. Geralis, A. Kyriakis, D. Loukas, G. Paspalaki, A. Stakia, M. Diamantopoulou, D. Karasavvas, G. Karathanasis, P. Kontaxakis, C. K. Koraka, A. Manousakis-katsikakis, A. Panagiotou, I. Papavergou, N. Saoulidou, K. Theofilatos, K. Vellidis, E. Vourliotis, G. Bakas, K. Kousouris, I. Papakrivopoulos, G. Tsipolitis, A. Zacharopoulou, I. Evangelou, C. Foudas, P. Gianneios, P. Katsoulis, P. Kokkas, S. Mallios, K. Manitara, N. Manthos, I. Papadopoulos, J. Strologas, M. Bartók, R. Chudasama, M. Csanad, M. M. A. Gadallah, S. Lökös, P. Major, K. Mandal, A. Mehta, G. Pasztor, O. Surányi, G. I. Veres, G. Bencze, C. Hajdu, D. Horvath, F. Sikler, V. Veszpremi, G. Vesztergombi, S. Czellar, J. Karancsi, J. Molnar, Z. Szillasi, D. Teyssier, P. Raics, Z. L. Trocsanyi, B. Ujvari, T. Csorgo, F. Nemes, T. Novak, S. Choudhury, J. R. Komaragiri, D. Kumar, L. Panwar, P. C. Tiwari, S. Bahinipati, D. Dash, C. Kar, P. Mal, T. Mishra, V. K. Muraleedharan Nair Bindhu, A. Nayak, D. K. Sahoo, N. Sur, S. K. Swain, S. Bansal, S. B. Beri, V. Bhatnagar, S. Chauhan, N. Dhingra, R. Gupta, A. Kaur, S. Kaur, P. Kumari, M. Lohan, M. Meena, K. Sandeep, S. Sharma, J. B. Singh, A. K. Virdi, A. Ahmed, A. Bhardwaj, B. C. Choudhary, R. B. Garg, M. Gola, S. Keshri, A. Kumar, M. Naimuddin, P. Priyanka, K. Ranjan, A. Shah, M. Bharti, R. Bhattacharya, S. Bhattacharya, D. Bhowmik, S. Dutta, S. Ghosh, B. Gomber, M. Maity, S. Nandan, P. Palit, A. Purohit, P. K. Rout, G. Saha, S. Sarkar, M. Sharan, B. Singh, S. Thakur, P. K. Behera, S. C. Behera, P. Kalbhor, A. Muhammad, R. Pradhan, P. R. Pujahari, A. Sharma, A. K. Sikdar, D. Dutta, V. Jha, V. Kumar, D. K. Mishra, K. Naskar, P. K. Netrakanti, L. M. Pant, P. Shukla, T. Aziz, M. A. Bhat, S. Dugad, R. Kumar Verma, U. Sarkar, S. Banerjee, S. Bhattacharya, S. Chatterjee, P. Das, M. Guchait, S. Karmakar, S. Kumar, G. Majumder, K. Mazumdar, S. Mukherjee, D. Roy, N. Sahoo, S. Dube, B. Kansal, A. Kapoor, K. Kothekar, S. Pandey, A. Rane, A. Rastogi, S. Sharma, H. Bakhshiansohi, S. Chenarani, S. M. Etesami, M. Khakzad, M. Mohammadi Najafabadi, M. Felcini, M. Grunewald, M. Abbrescia, R. Aly, C. Aruta, A. Colaleo, D. Creanza, N. De Filippis, M. De Palma, A. Di Florio, A. Di Pilato, W. Elmetenawee, L. Fiore, A. Gelmi, M. Gul, G. Iaselli, M. Ince, S. Lezki, G. Maggi, M. Maggi, I. Margjeka, J. A. Merlin, S. My, S. Nuzzo, A. Pompili, G. Pugliese, A. Ranieri, G. Selvaggi, L. Silvestris, F. M. Simone, R. Venditti, P. Verwilligen, G. Abbiendi, C. Battilana, D. Bonacorsi, L. Borgonovi, S. Braibant-Giacomelli, L. Brigliadori, R. Campanini, P. Capiluppi, A. Castro, F. R. Cavallo, M. Cuffiani, G. M. Dallavalle, T. Diotalevi, F. Fabbri, A. Fanfani, E. Fontanesi, P. Giacomelli, L. Giommi, C. Grandi, L. Guiducci, F. Iemmi, S. Lo Meo, S. Marcellini, G. Masetti, F. L. Navarria, A. Perrotta, F. Primavera, T. Rovelli, G. P. Siroli, N. Tosi, S. Albergo, S. Costa, A. Di Mattia, R. Potenza, A. Tricomi, C. Tuve, G. Barbagli, A. Cassese, R. Ceccarelli, V. Ciulli, C. Civinini, R. D’Alessandro, F. Fiori, E. Focardi, G. Latino, P. Lenzi, M. Lizzo, M. Meschini, S. Paoletti, R. Seidita, G. Sguazzoni, L. Viliani, L. Benussi, S. Bianco, D. Piccolo, M. Bozzo, F. Ferro, R. Mulargia, E. Robutti, S. Tosi, A. Benaglia, A. Beschi, F. Brivio, F. Cetorelli, V. Ciriolo, F. De Guio, M. E. Dinardo, P. Dini, S. Gennai, A. Ghezzi, P. Govoni, L. Guzzi, M. Malberti, S. Malvezzi, D. Menasce, F. Monti, L. Moroni, M. Paganoni, D. Pedrini, S. Ragazzi, T. Tabarelli de Fatis, D. Valsecchi, D. Zuolo, S. Buontempo, N. Cavallo, A. De Iorio, F. Fabozzi, F. Fienga, A. O. M. Iorio, L. Layer, L. Lista, S. Meola, P. Paolucci, B. Rossi, C. Sciacca, E. Voevodina, P. Azzi, N. Bacchetta, D. Bisello, A. Boletti, A. Bragagnolo, R. Carlin, P. Checchia, P. De Castro Manzano, T. Dorigo, F. Gasparini, U. Gasparini, S. Y. Hoh, M. Margoni, A. T. Meneguzzo, M. Presilla, P. Ronchese, R. Rossin, F. Simonetto, G. Strong, A. Tiko, M. Tosi, H. Yarar, M. Zanetti, P. Zotto, A. Zucchetta, A. Braghieri, S. Calzaferri, D. Fiorina, P. Montagna, S. P. Ratti, V. Re, M. Ressegotti, C. Riccardi, P. Salvini, I. Vai, P. Vitulo, M. Biasini, G. M. Bilei, D. Ciangottini, L. Fanò, P. Lariccia, G. Mantovani, V. Mariani, M. Menichelli, F. Moscatelli, A. Rossi, A. Santocchia, D. Spiga, T. Tedeschi, K. Androsov, P. Azzurri, G. Bagliesi, V. Bertacchi, L. Bianchini, T. Boccali, R. Castaldi, M. A. Ciocci, R. Dell’Orso, M. R. Di Domenico, S. Donato, L. Giannini, A. Giassi, M. T. Grippo, F. Ligabue, E. Manca, G. Mandorli, A. Messineo, F. Palla, G. Ramirez-Sanchez, A. Rizzi, G. Rolandi, S. Roy Chowdhury, A. Scribano, N. Shafiei, P. Spagnolo, R. Tenchini, G. Tonelli, N. Turini, A. Venturi, P. G. Verdini, F. Cavallari, M. Cipriani, D. Del Re, E. Di Marco, M. Diemoz, E. Longo, P. Meridiani, G. Organtini, F. Pandolfi, R. Paramatti, C. Quaranta, S. Rahatlou, C. Rovelli, F. Santanastasio, L. Soffi, R. Tramontano, N. Amapane, R. Arcidiacono, S. Argiro, M. Arneodo, N. Bartosik, R. Bellan, A. Bellora, C. Biino, A. Cappati, N. Cartiglia, S. Cometti, M. Costa, R. Covarelli, N. Demaria, B. Kiani, F. Legger, C. Mariotti, S. Maselli, E. Migliore, V. Monaco, E. Monteil, M. Monteno, M. M. Obertino, G. Ortona, L. Pacher, N. Pastrone, M. Pelliccioni, G. L. Pinna Angioni, M. Ruspa, R. Salvatico, F. Siviero, V. Sola, A. Solano, D. Soldi, A. Staiano, D. Trocino, S. Belforte, V. Candelise, M. Casarsa, F. Cossutti, A. Da Rold, G. Della Ricca, F. Vazzoler, S. Dogra, C. Huh, B. Kim, D. H. Kim, G. N. Kim, J. Lee, S. W. Lee, C. S. Moon, Y. D. Oh, S. I. Pak, B. C. Radburn-Smith, S. Sekmen, Y. C. Yang, H. Kim, D. H. Moon, B. Francois, T. J. Kim, J. Park, S. Cho, S. Choi, Y. Go, S. Ha, B. Hong, K. Lee, K. S. Lee, J. Lim, J. Park, S. K. Park, J. Yoo, J. Goh, A. Gurtu, H. S. Kim, Y. Kim, J. Almond, J. H. Bhyun, J. Choi, S. Jeon, J. Kim, J. S. Kim, S. Ko, H. Kwon, H. Lee, K. Lee, S. Lee, K. Nam, B. H. Oh, M. Oh, S. B. Oh, H. Seo, U. K. Yang, I. Yoon, D. Jeon, J. H. Kim, B. Ko, J. S. H. Lee, I. C. Park, Y. Roh, D. Song, I. J. Watson, H. D. Yoo, Y. Choi, C. Hwang, Y. Jeong, H. Lee, Y. Lee, I. Yu, Y. Maghrbi, V. Veckalns, A. Juodagalvis, A. Rinkevicius, G. Tamulaitis, W. A. T. Wan Abdullah, M. N. Yusli, Z. Zolkapli, J. F. Benitez, A. Castaneda Hernandez, J. A. Murillo Quijada, L. Valencia Palomo, H. Castilla-Valdez, E. De La Cruz-Burelo, I. Heredia-De La Cruz, R. Lopez-Fernandez, A. Sanchez-Hernandez, S. Carrillo Moreno, C. Oropeza Barrera, M. Ramirez-Garcia, F. Vazquez Valencia, J. Eysermans, I. Pedraza, H. A. Salazar Ibarguen, C. Uribe Estrada, A. Morelos Pineda, J. Mijuskovic, N. Raicevic, D. Krofcheck, S. Bheesette, P. H. Butler, A. Ahmad, M. I. Asghar, M. I. M. Awan, Q. Hassan, H. R. Hoorani, W. A. Khan, M. A. Shah, M. Shoaib, M. Waqas, V. Avati, L. Grzanka, M. Malawski, H. Bialkowska, M. Bluj, B. Boimska, T. Frueboes, M. Górski, M. Kazana, M. Szleper, P. Traczyk, P. Zalewski, K. Bunkowski, A. Byszuk, K. Doroba, A. Kalinowski, M. Konecki, J. Krolikowski, M. Olszewski, M. Walczak, M. Araujo, P. Bargassa, D. Bastos, P. Faccioli, M. Gallinaro, J. Hollar, N. Leonardo, T. Niknejad, J. Seixas, K. Shchelina, O. Toldaiev, J. Varela, S. Afanasiev, V. Alexakhin, P. Bunin, M. Gavrilenko, I. Golutvin, I. Gorbunov, V. Karjavine, A. Lanev, A. Malakhov, V. Matveev, V. V. Mitsyn, P. Moisenz, V. Palichik, V. Perelygin, M. Savina, S. Shmatov, S. Shulha, V. Smirnov, O. Teryaev, V. Trofimov, N. Voytishin, B. S. Yuldashev, A. Zarubin, G. Gavrilov, V. Golovtcov, Y. Ivanov, V. Kim, E. Kuznetsova, V. Murzin, V. Oreshkin, I. Smirnov, D. Sosnov, V. Sulimov, L. Uvarov, S. Volkov, A. Vorobyev, Yu. Andreev, A. Dermenev, S. Gninenko, N. Golubev, A. Karneyeu, M. Kirsanov, N. Krasnikov, A. Pashenkov, G. Pivovarov, D. Tlisov, A. Toropin, V. Epshteyn, V. Gavrilov, N. Lychkovskaya, A. Nikitenko, V. Popov, I. Pozdnyakov, G. Safronov, A. Spiridonov, A. Stepennov, M. Toms, E. Vlasov, A. Zhokin, T. Aushev, M. Chadeeva, A. Oskin, P. Parygin, S. Polikarpov, E. Zhemchugov, V. Andreev, M. Azarkin, I. Dremin, M. Kirakosyan, A. Terkulov, A. Belyaev, E. Boos, V. Bunichev, M. Dubinin, L. Dudko, V. Klyukhin, O. Kodolova, I. Lokhtin, S. Obraztsov, M. Perfilov, S. Petrushanko, V. Savrin, A. Snigirev, V. Blinov, T. Dimova, L. Kardapoltsev, I. Ovtin, Y. Skovpen, I. Azhgirey, I. Bayshev, V. Kachanov, A. Kalinin, D. Konstantinov, V. Petrov, R. Ryutin, A. Sobol, S. Troshin, N. Tyurin, A. Uzunian, A. Volkov, A. Babaev, A. Iuzhakov, V. Okhotnikov, L. Sukhikh, V. Borchsh, V. Ivanchenko, E. Tcherniaev, P. Adzic, P. Cirkovic, M. Dordevic, P. Milenovic, J. Milosevic, M. Aguilar-Benitez, J. Alcaraz Maestre, A. Álvarez Fernández, I. Bachiller, M. Barrio Luna, C. F. Bedoya, J. A. Brochero Cifuentes, C. A. Carrillo Montoya, M. Cepeda, M. Cerrada, N. Colino, B. De La Cruz, A. Delgado Peris, J. P. Fernández Ramos, J. Flix, M. C. Fouz, A. García Alonso, O. Gonzalez Lopez, S. Goy Lopez, J. M. Hernandez, M. I. Josa, J. León Holgado, D. Moran, Á. Navarro Tobar, A. Pérez-Calero Yzquierdo, J. Puerta Pelayo, I. Redondo, L. Romero, S. Sánchez Navas, M. S. Soares, A. Triossi, L. Urda Gómez, C. Willmott, C. Albajar, J. F. de Trocóniz, R. Reyes-Almanza, B. Alvarez Gonzalez, J. Cuevas, C. Erice, J. Fernandez Menendez, S. Folgueras, I. Gonzalez Caballero, E. Palencia Cortezon, C. Ramón Álvarez, J. Ripoll Sau, V. Rodríguez Bouza, S. Sanchez Cruz, A. Trapote, I. J. Cabrillo, A. Calderon, B. Chazin Quero, J. Duarte Campderros, M. Fernandez, P. J. Fernández Manteca, G. Gomez, C. Martinez Rivero, P. Martinez Ruiz del Arbol, F. Matorras, J. Piedra Gomez, C. Prieels, F. Ricci-Tam, T. Rodrigo, A. Ruiz-Jimeno, L. Russo, L. Scodellaro, I. Vila, J. M. Vizan Garcia, MK Jayananda, B. Kailasapathy, D. U. J. Sonnadara, D. D. C. Wickramarathna, W. G. D. Dharmaratna, K. Liyanage, N. Perera, N. Wickramage, T. K. Aarrestad, D. Abbaneo, B. Akgun, E. Auffray, G. Auzinger, J. Baechler, P. Baillon, A. H. Ball, D. Barney, J. Bendavid, N. Beni, M. Bianco, A. Bocci, P. Bortignon, E. Bossini, E. Brondolin, T. Camporesi, G. Cerminara, L. Cristella, D. d’Enterria, A. Dabrowski, N. Daci, V. Daponte, A. David, A. De Roeck, M. Deile, R. Di Maria, M. Dobson, M. Dünser, N. Dupont, A. Elliott-Peisert, N. Emriskova, F. Fallavollita, D. Fasanella, S. Fiorendi, G. Franzoni, J. Fulcher, W. Funk, S. Giani, D. Gigi, K. Gill, F. Glege, L. Gouskos, M. Guilbaud, D. Gulhan, M. Haranko, J. Hegeman, Y. Iiyama, V. Innocente, T. James, P. Janot, J. Kaspar, J. Kieseler, M. Komm, N. Kratochwil, C. Lange, P. Lecoq, K. Long, C. Lourenço, L. Malgeri, M. Mannelli, A. Massironi, F. Meijers, S. Mersi, E. Meschi, F. Moortgat, M. Mulders, J. Ngadiuba, J. Niedziela, S. Orfanelli, L. Orsini, F. Pantaleo, L. Pape, E. Perez, M. Peruzzi, A. Petrilli, G. Petrucciani, A. Pfeiffer, M. Pierini, D. Rabady, A. Racz, M. Rieger, M. Rovere, H. Sakulin, J. Salfeld-Nebgen, S. Scarfi, C. Schäfer, C. Schwick, M. Selvaggi, A. Sharma, P. Silva, W. Snoeys, P. Sphicas, J. Steggemann, S. Summers, V. R. Tavolaro, D. Treille, A. Tsirou, G. P. Van Onsem, A. Vartak, M. Verzetti, K. A. Wozniak, W. D. Zeuner, L. Caminada, W. Erdmann, R. Horisberger, Q. Ingram, H. C. Kaestli, D. Kotlinski, U. Langenegger, T. Rohe, M. Backhaus, P. Berger, A. Calandri, N. Chernyavskaya, G. Dissertori, M. Dittmar, M. Donegà, C. Dorfer, T. Gadek, T. A. Gómez Espinosa, C. Grab, D. Hits, W. Lustermann, A.-M. Lyon, R. A. Manzoni, M. T. Meinhard, F. Micheli, F. Nessi-Tedaldi, F. Pauss, V. Perovic, G. Perrin, L. Perrozzi, S. Pigazzini, M. G. Ratti, M. Reichmann, C. Reissel, T. Reitenspiess, B. Ristic, D. Ruini, D. A. Sanz Becerra, M. Schönenberger, L. Shchutska, V. Stampf, M. L. Vesterbacka Olsson, R. Wallny, D. H. Zhu, C. Amsler, C. Botta, D. Brzhechko, M. F. Canelli, A. De Cosa, R. Del Burgo, J. K. Heikkilä, M. Huwiler, A. Jofrehei, B. Kilminster, S. Leontsinis, A. Macchiolo, P. Meiring, V. M. Mikuni, U. Molinatti, I. Neutelings, G. Rauco, A. Reimers, P. Robmann, K. Schweiger, Y. Takahashi, S. Wertz, C. Adloff, C. M. Kuo, W. Lin, A. Roy, T. Sarkar, S. S. Yu, L. Ceard, P. Chang, Y. Chao, K. F. Chen, P. H. Chen, W.-S. Hou, Y. Y. Li, R.-S. Lu, E. Paganis, A. Psallidas, A. Steen, E. Yazgan, B. Asavapibhop, C. Asawatangtrakuldee, N. Srimanobhas, F. Boran, S. Damarseckin, Z. S. Demiroglu, F. Dolek, C. Dozen, I. Dumanoglu, E. Eskut, G. Gokbulut, Y. Guler, E. Gurpinar Guler, I. Hos, C. Isik, E. E. Kangal, O. Kara, A. Kayis Topaksu, U. Kiminsu, G. Onengut, K. Ozdemir, A. Polatoz, A. E. Simsek, B. Tali, U. G. Tok, S. Turkcapar, I. S. Zorbakir, C. Zorbilmez, B. Isildak, G. Karapinar, K. Ocalan, M. Yalvac, I. O. Atakisi, E. Gülmez, M. Kaya, O. Kaya, Ö. Özçelik, S. Tekten, E. A. Yetkin, A. Cakir, K. Cankocak, Y. Komurcu, S. Sen, F. Aydogmus Sen, S. Cerci, B. Kaynak, S. Ozkorucuklu, D. Sunar Cerci, B. Grynyov, L. Levchuk, E. Bhal, S. Bologna, J. J. Brooke, E. Clement, D. Cussans, H. Flacher, J. Goldstein, G. P. Heath, H. F. Heath, L. Kreczko, B. Krikler, S. Paramesvaran, T. Sakuma, S. Seif El Nasr-Storey, V. J. Smith, J. Taylor, A. Titterton, K. W. Bell, A. Belyaev, C. Brew, R. M. Brown, D. J. A. Cockerill, K. V. Ellis, K. Harder, S. Harper, J. Linacre, K. Manolopoulos, D. M. Newbold, E. Olaiya, D. Petyt, T. Reis, T. Schuh, C. H. Shepherd-Themistocleous, A. Thea, I. R. Tomalin, T. Williams, R. Bainbridge, P. Bloch, S. Bonomally, J. Borg, S. Breeze, O. Buchmuller, A. Bundock, V. Cepaitis, G. S. Chahal, D. Colling, P. Dauncey, G. Davies, M. Della Negra, P. Everaerts, G. Fedi, G. Hall, G. Iles, J. Langford, L. Lyons, A.-M. Magnan, S. Malik, A. Martelli, V. Milosevic, J. Nash, V. Palladino, M. Pesaresi, D. M. Raymond, A. Richards, A. Rose, E. Scott, C. Seez, A. Shtipliyski, M. Stoye, A. Tapper, K. Uchida, T. Virdee, N. Wardle, S. N. Webb, D. Winterbottom, A. G. Zecchinelli, S. C. Zenz, J. E. Cole, P. R. Hobson, A. Khan, P. Kyberd, C. K. Mackay, I. D. Reid, L. Teodorescu, S. Zahid, A. Brinkerhoff, K. Call, B. Caraway, J. Dittmann, K. Hatakeyama, A. R. Kanuganti, C. Madrid, B. McMaster, N. Pastika, S. Sawant, C. Smith, R. Bartek, A. Dominguez, R. Uniyal, A. M. Vargas Hernandez, A. Buccilli, O. Charaf, S. I. Cooper, S. V. Gleyzer, C. Henderson, P. Rumerio, C. West, A. Akpinar, A. Albert, D. Arcaro, C. Cosby, Z. Demiragli, D. Gastler, C. Richardson, J. Rohlf, K. Salyer, D. Sperka, D. Spitzbart, I. Suarez, S. Yuan, D. Zou, G. Benelli, B. Burkle, X. Coubez, D. Cutts, Y. t. Duh, M. Hadley, U. Heintz, J. M. Hogan, K. H. M. Kwok, E. Laird, G. Landsberg, K. T. Lau, J. Lee, M. Narain, S. Sagir, R. Syarif, E. Usai, W. Y. Wong, D. Yu, W. Zhang, R. Band, C. Brainerd, R. Breedon, M. Calderon De La Barca Sanchez, M. Chertok, J. Conway, R. Conway, P. T. Cox, R. Erbacher, C. Flores, G. Funk, F. Jensen, W. Ko, O. Kukral, R. Lander, M. Mulhearn, D. Pellett, J. Pilot, M. Shi, D. Taylor, K. Tos, M. Tripathi, Y. Yao, F. Zhang, M. Bachtis, R. Cousins, A. Dasgupta, A. Florent, D. Hamilton, J. Hauser, M. Ignatenko, T. Lam, N. Mccoll, W. A. Nash, S. Regnard, D. Saltzberg, C. Schnaible, B. Stone, V. Valuev, K. Burt, Y. Chen, R. Clare, J. W. Gary, S. M. A. Ghiasi Shirazi, G. Hanson, G. Karapostoli, O. R. Long, N. Manganelli, M. Olmedo Negrete, M. I. Paneva, W. Si, S. Wimpenny, Y. Zhang, J. G. Branson, P. Chang, S. Cittolin, S. Cooperstein, N. Deelen, M. Derdzinski, J. Duarte, R. Gerosa, D. Gilbert, B. Hashemi, D. Klein, V. Krutelyov, J. Letts, M. Masciovecchio, S. May, S. Padhi, M. Pieri, V. Sharma, M. Tadel, F. Würthwein, A. Yagil, N. Amin, C. Campagnari, M. Citron, A. Dorsett, V. Dutta, J. Incandela, B. Marsh, H. Mei, A. Ovcharova, H. Qu, M. Quinnan, J. Richman, U. Sarica, D. Stuart, S. Wang, D. Anderson, A. Bornheim, O. Cerri, I. Dutta, J. M. Lawhorn, N. Lu, J. Mao, H. B. Newman, T. Q. Nguyen, J. Pata, M. Spiropulu, J. R. Vlimant, S. Xie, Z. Zhang, R. Y. Zhu, J. Alison, M. B. Andrews, T. Ferguson, T. Mudholkar, M. Paulini, M. Sun, I. Vorobiev, J. P. Cumalat, W. T. Ford, E. MacDonald, T. Mulholland, R. Patel, A. Perloff, K. Stenson, K. A. Ulmer, S. R. Wagner, J. Alexander, Y. Cheng, J. Chu, D. J. Cranshaw, A. Datta, A. Frankenthal, K. Mcdermott, J. Monroy, J. R. Patterson, D. Quach, A. Ryd, W. Sun, S. M. Tan, Z. Tao, J. Thom, P. Wittich, M. Zientek, S. Abdullin, M. Albrow, M. Alyari, G. Apollinari, A. Apresyan, A. Apyan, S. Banerjee, L. A. T. Bauerdick, A. Beretvas, D. Berry, J. Berryhill, P. C. Bhat, K. Burkett, J. N. Butler, A. Canepa, G. B. Cerati, H. W. K. Cheung, F. Chlebana, M. Cremonesi, V. D. Elvira, J. Freeman, Z. Gecse, E. Gottschalk, L. Gray, D. Green, S. Grünendahl, O. Gutsche, R. M. Harris, S. Hasegawa, R. Heller, T. C. Herwig, J. Hirschauer, B. Jayatilaka, S. Jindariani, M. Johnson, U. Joshi, P. Klabbers, T. Klijnsma, B. Klima, M. J. Kortelainen, S. Lammel, D. Lincoln, R. Lipton, M. Liu, T. Liu, J. Lykken, K. Maeshima, D. Mason, P. McBride, P. Merkel, S. Mrenna, S. Nahn, V. O’Dell, V. Papadimitriou, K. Pedro, C. Pena, O. Prokofyev, F. Ravera, A. Reinsvold Hall, L. Ristori, B. Schneider, E. Sexton-Kennedy, N. Smith, A. Soha, W. J. Spalding, L. Spiegel, S. Stoynev, J. Strait, L. Taylor, S. Tkaczyk, N. V. Tran, L. Uplegger, E. W. Vaandering, H. A. Weber, A. Woodard, D. Acosta, P. Avery, D. Bourilkov, L. Cadamuro, V. Cherepanov, F. Errico, R. D. Field, D. Guerrero, B. M. Joshi, M. Kim, J. Konigsberg, A. Korytov, K. H. Lo, K. Matchev, N. Menendez, G. Mitselmakher, D. Rosenzweig, K. Shi, J. Wang, S. Wang, X. Zuo, T. Adams, A. Askew, D. Diaz, R. Habibullah, S. Hagopian, V. Hagopian, K. F. Johnson, R. Khurana, T. Kolberg, G. Martinez, H. Prosper, C. Schiber, R. Yohay, J. Zhang, M. M. Baarmand, S. Butalla, T. Elkafrawy, M. Hohlmann, D. Noonan, M. Rahmani, M. Saunders, F. Yumiceva, M. R. Adams, L. Apanasevich, H. Becerril Gonzalez, R. Cavanaugh, X. Chen, S. Dittmer, O. Evdokimov, C. E. Gerber, D. A. Hangal, D. J. Hofman, C. Mills, G. Oh, T. Roy, M. B. Tonjes, N. Varelas, J. Viinikainen, X. Wang, Z. Wu, M. Alhusseini, K. Dilsiz, S. Durgut, R. P. Gandrajula, M. Haytmyradov, V. Khristenko, O. K. Köseyan, J.-P. Merlo, A. Mestvirishvili, A. Moeller, J. Nachtman, H. Ogul, Y. Onel, F. Ozok, A. Penzo, C. Snyder, E. Tiras, J. Wetzel, K. Yi, O. Amram, B. Blumenfeld, L. Corcodilos, M. Eminizer, A. V. Gritsan, S. Kyriacou, P. Maksimovic, C. Mantilla, J. Roskes, M. Swartz, T. Á. Vámi, C. Baldenegro Barrera, P. Baringer, A. Bean, A. Bylinkin, T. Isidori, S. Khalil, J. King, G. Krintiras, A. Kropivnitskaya, C. Lindsey, N. Minafra, M. Murray, C. Rogan, C. Royon, S. Sanders, E. Schmitz, J. D. Tapia Takaki, Q. Wang, J. Williams, G. Wilson, S. Duric, A. Ivanov, K. Kaadze, D. Kim, Y. Maravin, T. Mitchell, A. Modak, A. Mohammadi, F. Rebassoo, D. Wright, E. Adams, A. Baden, O. Baron, A. Belloni, S. C. Eno, Y. Feng, N. J. Hadley, S. Jabeen, G. Y. Jeng, R. G. Kellogg, T. Koeth, A. C. Mignerey, S. Nabili, M. Seidel, A. Skuja, S. C. Tonwar, L. Wang, K. Wong, D. Abercrombie, B. Allen, R. Bi, S. Brandt, W. Busza, I. A. Cali, Y. Chen, M. D’Alfonso, G. Gomez Ceballos, M. Goncharov, P. Harris, D. Hsu, M. Hu, M. Klute, D. Kovalskyi, J. Krupa, Y.-J. Lee, P. D. Luckey, B. Maier, A. C. Marini, C. Mcginn, C. Mironov, S. Narayanan, X. Niu, C. Paus, D. Rankin, C. Roland, G. Roland, Z. Shi, G. S. F. Stephans, K. Sumorok, K. Tatar, D. Velicanu, J. Wang, T. W. Wang, Z. Wang, B. Wyslouch, R. M. Chatterjee, A. Evans, S. Guts, P. Hansen, J. Hiltbrand, Sh. Jain, M. Krohn, Y. Kubota, Z. Lesko, J. Mans, M. Revering, R. Rusack, R. Saradhy, N. Schroeder, N. Strobbe, M. A. Wadud, J. G. Acosta, S. Oliveros, K. Bloom, S. Chauhan, D. R. Claes, C. Fangmeier, L. Finco, F. Golf, J. R. González Fernández, I. Kravchenko, J. E. Siado, G. R. Snow, B. Stieger, W. Tabb, F. Yan, G. Agarwal, C. Harrington, L. Hay, I. Iashvili, A. Kharchilava, C. McLean, D. Nguyen, A. Parker, J. Pekkanen, S. Rappoccio, B. Roozbahani, G. Alverson, E. Barberis, C. Freer, Y. Haddad, A. Hortiangtham, G. Madigan, B. Marzocchi, D. M. Morse, V. Nguyen, T. Orimoto, L. Skinnari, A. Tishelman-Charny, T. Wamorkar, B. Wang, A. Wisecarver, D. Wood, S. Bhattacharya, J. Bueghly, Z. Chen, A. Gilbert, T. Gunter, K. A. Hahn, N. Odell, M. H. Schmitt, K. Sung, M. Velasco, R. Bucci, N. Dev, R. Goldouzian, M. Hildreth, K. Hurtado Anampa, C. Jessop, D. J. Karmgard, K. Lannon, W. Li, N. Loukas, N. Marinelli, I. Mcalister, F. Meng, K. Mohrman, Y. Musienko, R. Ruchti, P. Siddireddy, S. Taroni, M. Wayne, A. Wightman, M. Wolf, L. Zygala, J. Alimena, B. Bylsma, B. Cardwell, L. S. Durkin, B. Francis, C. Hill, A. Lefeld, B. L. Winer, B. R. Yates, G. Dezoort, P. Elmer, B. Greenberg, N. Haubrich, S. Higginbotham, A. Kalogeropoulos, G. Kopp, S. Kwan, D. Lange, M. T. Lucchini, J. Luo, D. Marlow, K. Mei, I. Ojalvo, J. Olsen, C. Palmer, P. Piroué, D. Stickland, C. Tully, S. Malik, S. Norberg, V. E. Barnes, R. Chawla, S. Das, L. Gutay, M. Jones, A. W. Jung, B. Mahakud, G. Negro, N. Neumeister, C. C. Peng, S. Piperov, H. Qiu, J. F. Schulte, N. Trevisani, F. Wang, R. Xiao, W. Xie, T. Cheng, J. Dolen, N. Parashar, M. Stojanovic, A. Baty, S. Dildick, K. M. Ecklund, S. Freed, F. J. M. Geurts, M. Kilpatrick, A. Kumar, W. Li, B. P. Padley, R. Redjimi, J. Roberts, J. Rorie, W. Shi, A. G. Stahl Leiton, A. Zhang, A. Bodek, P. de Barbaro, R. Demina, J. L. Dulemba, C. Fallon, T. Ferbel, M. Galanti, A. Garcia-Bellido, O. Hindrichs, A. Khukhunaishvili, E. Ranken, R. Taus, B. Chiarito, J. P. Chou, A. Gandrakota, Y. Gershtein, E. Halkiadakis, A. Hart, M. Heindl, E. Hughes, S. Kaplan, O. Karacheban, I. Laflotte, A. Lath, R. Montalvo, K. Nash, M. Osherson, S. Salur, S. Schnetzer, S. Somalwar, R. Stone, S. A. Thayil, S. Thomas, H. Wang, H. Acharya, A. G. Delannoy, S. Spanier, O. Bouhali, M. Dalchenko, A. Delgado, R. Eusebi, J. Gilmore, T. Huang, T. Kamon, H. Kim, S. Luo, S. Malhotra, R. Mueller, D. Overton, L. Perniè, D. Rathjens, A. Safonov, J. Sturdy, N. Akchurin, J. Damgov, V. Hegde, S. Kunori, K. Lamichhane, S. W. Lee, T. Mengke, S. Muthumuni, T. Peltola, S. Undleeb, I. Volobouev, Z. Wang, A. Whitbeck, E. Appelt, S. Greene, A. Gurrola, R. Janjam, W. Johns, C. Maguire, A. Melo, H. Ni, K. Padeken, F. Romeo, P. Sheldon, S. Tuo, J. Velkovska, M. Verweij, L. Ang, M. W. Arenton, B. Cox, G. Cummings, J. Hakala, R. Hirosky, M. Joyce, A. Ledovskoy, C. Neu, B. Tannenwald, Y. Wang, E. Wolfe, F. Xia, P. E. Karchin, N. Poudyal, P. Thapa, K. Black, T. Bose, J. Buchanan, C. Caillol, S. Dasu, I. De Bruyn, C. Galloni, H. He, M. Herndon, A. Hervé, U. Hussain, A. Lanaro, A. Loeliger, R. Loveless, J. Madhusudanan Sreekala, A. Mallampalli, D. Pinna, T. Ruggles, A. Savin, V. Shang, V. Sharma, W. H. Smith, D. Teague, S. Trembath-reichert, W. Vetens, CMS Collaboration

**Affiliations:** 1 Yerevan Physics Institute, Yerevan, Armenia; 2 Institut für Hochenergiephysik, Vienna, Austria; 3 Institute for Nuclear Problems, Minsk, Belarus; 4 Universiteit Antwerpen, Antwerpen, Belgium; 5 Vrije Universiteit Brussel, Brussels, Belgium; 6 Université Libre de Bruxelles, Brussels, Belgium; 7 Ghent University, Ghent, Belgium; 8 Université Catholique de Louvain, Louvain-la-Neuve, Belgium; 9 Centro Brasileiro de Pesquisas Fisicas, Rio de Janeiro, Brazil; 10 Universidade do Estado do Rio de Janeiro, Rio de Janeiro, Brazil; 11 Universidade Estadual Paulista, Universidade Federal do ABC, São Paulo, Brazil; 12 Institute for Nuclear Research and Nuclear Energy, Bulgarian Academy of Sciences, Sofia, Bulgaria; 13 University of Sofia, Sofia, Bulgaria; 14 Beihang University, Beijing, China; 15 Department of Physics, Tsinghua University, Beijing, China; 16 Institute of High Energy Physics, Beijing, China; 17 State Key Laboratory of Nuclear Physics and Technology, Peking University, Beijing, China; 18 Sun Yat-Sen University, Guangzhou, China; 19 Institute of Modern Physics and Key Laboratory of Nuclear Physics and Ion-beam Application (MOE)-Fudan University, Shanghai, China; 20 Zhejiang University, Hangzhou, China; 21 Universidad de Los Andes, Bogotá, Colombia; 22 Universidad de Antioquia, Medellin, Colombia; 23 University of Split, Faculty of Electrical Engineering, Mechanical Engineering and Naval Architecture, Split, Croatia; 24 University of Split, Faculty of Science, Split, Croatia; 25 Institute Rudjer Boskovic, Zagreb, Croatia; 26 University of Cyprus, Nicosia, Cyprus; 27 Charles University, Prague, Czech Republic; 28 Escuela Politecnica Nacional, Quito, Ecuador; 29 Universidad San Francisco de Quito, Quito, Ecuador; 30 Academy of Scientific Research and Technology of the Arab Republic of Egypt, Egyptian Network of High Energy Physics, Cairo, Egypt; 31 Center for High Energy Physics (CHEP-FU), Fayoum University, El-Fayoum, Egypt; 32 National Institute of Chemical Physics and Biophysics, Tallinn, Estonia; 33 Department of Physics, University of Helsinki, Helsinki, Finland; 34 Helsinki Institute of Physics, Helsinki, Finland; 35 Lappeenranta University of Technology, Lappeenranta, Finland; 36 IRFU, CEA, Université Paris-Saclay, Gif-sur-Yvette, France; 37 Laboratoire Leprince-Ringuet, CNRS/IN2P3, Ecole Polytechnique, Institut Polytechnique de Paris, Palaiseau, France; 38 Université de Strasbourg, CNRS, IPHC UMR 7178, Strasbourg, France; 39 Université de Lyon, Université Claude Bernard Lyon 1, CNRS-IN2P3, Institut de Physique Nucléaire de Lyon, Villeurbanne, France; 40 Georgian Technical University, Tbilisi, Georgia; 41 RWTH Aachen University, I. Physikalisches Institut, Aachen, Germany; 42 RWTH Aachen University, III. Physikalisches Institut A, Aachen, Germany; 43 RWTH Aachen University, III. Physikalisches Institut B, Aachen, Germany; 44 Deutsches Elektronen-Synchrotron, Hamburg, Germany; 45 University of Hamburg, Hamburg, Germany; 46 Karlsruher Institut fuer Technologie, Karlsruhe, Germany; 47 Institute of Nuclear and Particle Physics (INPP), NCSR Demokritos, Aghia Paraskevi, Greece; 48 National and Kapodistrian University of Athens, Athens, Greece; 49 National Technical University of Athens, Athens, Greece; 50 University of Ioánnina, Ioánnina, Greece; 51 MTA-ELTE Lendület CMS Particle and Nuclear Physics Group, Eötvös Loránd University, Budapest, Hungary; 52 Wigner Research Centre for Physics, Budapest, Hungary; 53 Institute of Nuclear Research ATOMKI, Debrecen, Hungary; 54 Institute of Physics, University of Debrecen, Debrecen, Hungary; 55 Eszterhazy Karoly University, Karoly Robert Campus, Gyongyos, Hungary; 56 Indian Institute of Science (IISc), Bangalore, India; 57 National Institute of Science Education and Research, HBNI, Bhubaneswar, India; 58 Panjab University, Chandigarh, India; 59 University of Delhi, Delhi, India; 60 Saha Institute of Nuclear Physics, HBNI, Kolkata, India; 61 Indian Institute of Technology Madras, Madras, India; 62 Bhabha Atomic Research Centre, Mumbai, India; 63 Tata Institute of Fundamental Research-A, Mumbai, India; 64 Tata Institute of Fundamental Research-B, Mumbai, India; 65 Indian Institute of Science Education and Research (IISER), Pune, India; 66 Department of Physics, Isfahan University of Technology, Isfahan, Iran; 67 Institute for Research in Fundamental Sciences (IPM), Tehran, Iran; 68 University College Dublin, Dublin, Ireland; 69 INFN Sezione di Bari, Università di Bari, Politecnico di Bari, Bari, Italy; 70 INFN Sezione di Bologna, Università di Bologna, Bologna, Italy; 71 INFN Sezione di Catania, Università di Catania, Catania, Italy; 72 INFN Sezione di Firenze, Università di Firenze, Florence, Italy; 73 INFN Laboratori Nazionali di Frascati, Frascati, Italy; 74 INFN Sezione di Genova, Università di Genova, Genoa, Italy; 75 INFN Sezione di Milano-Bicocca, Università di Milano-Bicocca, Milan, Italy; 76 INFN Sezione di Napoli, Università di Napoli ’Federico II’, Napoli, Italy, Università della Basilicata, Potenza, Italy, Università G. Marconi, Rome, Italy; 77 INFN Sezione di Padova, Università di Padova, Padova, Italy, Università di Trento, Trento, Italy; 78 INFN Sezione di Pavia, Università di Pavia, Pavia, Italy; 79 INFN Sezione di Perugia, Università di Perugia, Perugia, Italy; 80 INFN Sezione di Pisa, Università di Pisa, Scuola Normale Superiore di Pisa, Pisa, Italy; 81 INFN Sezione di Roma, Sapienza Università di Roma, Rome, Italy; 82 INFN Sezione di Torino, Università di Torino, Torino, Italy, Università del Piemonte Orientale, Novara, Italy; 83 INFN Sezione di Trieste, Università di Trieste, Trieste, Italy; 84 Kyungpook National University, Daegu, Korea; 85 Chonnam National University, Institute for Universe and Elementary Particles, Kwangju, Korea; 86 Hanyang University, Seoul, Korea; 87 Korea University, Seoul, Korea; 88 Kyung Hee University, Department of Physics, Seoul, Republic of Korea; 89 Sejong University, Seoul, Korea; 90 Seoul National University, Seoul, Korea; 91 University of Seoul, Seoul, Korea; 92 Yonsei University, Department of Physics, Seoul, Korea; 93 Sungkyunkwan University, Suwon, Korea; 94 College of Engineering and Technology, American University of the Middle East (AUM), Kuwait; 95 Riga Technical University, Riga, Latvia; 96 Vilnius University, Vilnius, Lithuania; 97 National Centre for Particle Physics, Universiti Malaya, Kuala Lumpur, Malaysia; 98 Universidad de Sonora (UNISON), Hermosillo, Mexico; 99 Centro de Investigacion y de Estudios Avanzados del IPN, Mexico City, Mexico; 100 Universidad Iberoamericana, Mexico City, Mexico; 101 Benemerita Universidad Autonoma de Puebla, Puebla, Mexico; 102 Universidad Autónoma de San Luis Potosí, San Luis Potosí, Mexico; 103 University of Montenegro, Podgorica, Montenegro; 104 University of Auckland, Auckland, New Zealand; 105 University of Canterbury, Christchurch, New Zealand; 106 National Centre for Physics, Quaid-I-Azam University, Islamabad, Pakistan; 107 AGH University of Science and Technology Faculty of Computer Science, Electronics and Telecommunications, Krakow, Poland; 108 National Centre for Nuclear Research, Swierk, Poland; 109 Institute of Experimental Physics, Faculty of Physics, University of Warsaw, Warsaw, Poland; 110 Laboratório de Instrumentação e Física Experimental de Partículas, Lisbon, Portugal; 111 Joint Institute for Nuclear Research, Dubna, Russia; 112 Petersburg Nuclear Physics Institute, Gatchina (St. Petersburg), Russia; 113 Institute for Nuclear Research, Moscow, Russia; 114 Institute for Theoretical and Experimental Physics named by A.I. Alikhanov of NRC ‘Kurchatov Institute’, Moscow, Russia; 115 Moscow Institute of Physics and Technology, Moscow, Russia; 116 National Research Nuclear University ’Moscow Engineering Physics Institute’ (MEPhI), Moscow, Russia; 117 P.N. Lebedev Physical Institute, Moscow, Russia; 118 Skobeltsyn Institute of Nuclear Physics, Lomonosov Moscow State University, Moscow, Russia; 119 Novosibirsk State University (NSU), Novosibirsk, Russia; 120 Institute for High Energy Physics of National Research Centre ‘Kurchatov Institute’, Protvino, Russia; 121 National Research Tomsk Polytechnic University, Tomsk, Russia; 122 Tomsk State University, Tomsk, Russia; 123 University of Belgrade: Faculty of Physics and VINCA Institute of Nuclear Sciences, Belgrade, Serbia; 124 Centro de Investigaciones Energéticas Medioambientales y Tecnológicas (CIEMAT), Madrid, Spain; 125 Universidad Autónoma de Madrid, Madrid, Spain; 126 Universidad de Oviedo, Instituto Universitario de Ciencias y Tecnologías Espaciales de Asturias (ICTEA), Oviedo, Spain; 127 Instituto de Física de Cantabria (IFCA), CSIC-Universidad de Cantabria, Santander, Spain; 128 University of Colombo, Colombo, Sri Lanka; 129 University of Ruhuna, Department of Physics, Matara, Sri Lanka; 130 CERN, European Organization for Nuclear Research, Geneva, Switzerland; 131 Paul Scherrer Institut, Villigen, Switzerland; 132 ETH Zurich-Institute for Particle Physics and Astrophysics (IPA), Zurich, Switzerland; 133 Universität Zürich, Zurich, Switzerland; 134 National Central University, Chung-Li, Taiwan; 135 National Taiwan University (NTU), Taipei, Taiwan; 136 Chulalongkorn University, Faculty of Science, Department of Physics, Bangkok, Thailand; 137 Çukurova University, Physics Department, Science and Art Faculty, Adana, Turkey; 138 Middle East Technical University, Physics Department, Ankara, Turkey; 139 Bogazici University, Istanbul, Turkey; 140 Istanbul Technical University, Istanbul, Turkey; 141 Istanbul University, Istanbul, Turkey; 142 Institute for Scintillation Materials of National Academy of Science of Ukraine, Kharkov, Ukraine; 143 National Scientific Center, Kharkov Institute of Physics and Technology, Kharkov, Ukraine; 144 University of Bristol, Bristol, UK; 145 Rutherford Appleton Laboratory, Didcot, UK; 146 Imperial College, London, UK; 147 Brunel University, Uxbridge, UK; 148 Baylor University, Waco, USA; 149 Catholic University of America, Washington, DC USA; 150 The University of Alabama, Tuscaloosa, USA; 151 Boston University, Boston, USA; 152 Brown University, Providence, USA; 153 University of California, Davis, Davis, USA; 154 University of California, Los Angeles, USA; 155 University of California, Riverside, Riverside, USA; 156 University of California, San Diego, La Jolla, USA; 157 Department of Physics, University of California, Santa Barbara, Santa Barbara, USA; 158 California Institute of Technology, Pasadena, USA; 159 Carnegie Mellon University, Pittsburgh, USA; 160 University of Colorado Boulder, Boulder, USA; 161 Cornell University, Ithaca, USA; 162 Fermi National Accelerator Laboratory, Batavia, USA; 163 University of Florida, Gainesville, USA; 164 Florida State University, Tallahassee, USA; 165 Florida Institute of Technology, Melbourne, USA; 166 University of Illinois at Chicago (UIC), Chicago, USA; 167 The University of Iowa, Iowa City, USA; 168 Johns Hopkins University, Baltimore, USA; 169 The University of Kansas, Lawrence, USA; 170 Kansas State University, Manhattan, USA; 171 Lawrence Livermore National Laboratory, Livermore, USA; 172 University of Maryland, College Park, USA; 173 Massachusetts Institute of Technology, Cambridge, USA; 174 University of Minnesota, Minneapolis, USA; 175 University of Mississippi, Oxford, USA; 176 University of Nebraska-Lincoln, Lincoln, USA; 177 State University of New York at Buffalo, Buffalo, USA; 178 Northeastern University, Boston, USA; 179 Northwestern University, Evanston, USA; 180 University of Notre Dame, Notre Dame, USA; 181 The Ohio State University, Columbus, USA; 182 Princeton University, Princeton, USA; 183 University of Puerto Rico, Mayaguez, USA; 184 Purdue University, West Lafayette, USA; 185 Purdue University Northwest, Hammond, USA; 186 Rice University, Houston, USA; 187 University of Rochester, Rochester, USA; 188 Rutgers, The State University of New Jersey, Piscataway, USA; 189 University of Tennessee, Knoxville, USA; 190 Texas A&M University, College Station, USA; 191 Texas Tech University, Lubbock, USA; 192 Vanderbilt University, Nashville, USA; 193 University of Virginia, Charlottesville, USA; 194 Wayne State University, Detroit, USA; 195 University of Wisconsin-Madison, Madison, WI USA; 196 Vienna University of Technology, Vienna, Austria; 197 Institute of Basic and Applied Sciences, Faculty of Engineering, Arab Academy for Science, Technology and Maritime Transport, Alexandria, Egypt, Alexandria, Egypt; 198 Université Libre de Bruxelles, Bruxelles, Belgium; 199 IRFU, CEA, Université Paris-Saclay, Gif-sur-Yvette, France; 200 Universidade Estadual de Campinas, Campinas, Brazil; 201 Federal University of Rio Grande do Sul, Porto Alegre, Brazil; 202 UFMS, Nova Andradina, Brazil; 203 Universidade Federal de Pelotas, Pelotas, Brazil; 204 University of Chinese Academy of Sciences, Beijing, China; 205 Institute for Theoretical and Experimental Physics named by A.I. Alikhanov of NRC ‘Kurchatov Institute’, Moscow, Russia; 206 Joint Institute for Nuclear Research, Dubna, Russia; 207 Cairo University, Cairo, Egypt; 208 Suez University, Suez, Egypt; 209 British University in Egypt, Cairo, Egypt; 210 Zewail City of Science and Technology, Zewail, Egypt; 211 Fayoum University, El-Fayoum, Egypt; 212 Purdue University, West Lafayette, USA; 213 Université de Haute Alsace, Mulhouse, France; 214 Tbilisi State University, Tbilisi, Georgia; 215 Erzincan Binali Yildirim University, Erzincan, Turkey; 216 CERN, European Organization for Nuclear Research, Geneva, Switzerland; 217 RWTH Aachen University, III. Physikalisches Institut A, Aachen, Germany; 218 University of Hamburg, Hamburg, Germany; 219 Department of Physics, Isfahan University of Technology, Isfahan, Iran, Isfahan, Iran; 220 Brandenburg University of Technology, Cottbus, Germany; 221 Skobeltsyn Institute of Nuclear Physics, Lomonosov Moscow State University, Moscow, Russia; 222 Institute of Physics, University of Debrecen, Debrecen, Hungary, Debrecen, Hungary; 223 Physics Department, Faculty of Science, Assiut University, Assiut, Egypt; 224 MTA-ELTE Lendület CMS Particle and Nuclear Physics Group, Eötvös Loránd University, Budapest, Hungary, Budapest, Hungary; 225 Institute of Nuclear Research ATOMKI, Debrecen, Hungary; 226 IIT Bhubaneswar, Bhubaneswar, India, Bhubaneswar, India; 227 Institute of Physics, Bhubaneswar, India; 228 G.H.G. Khalsa College, Punjab, India; 229 Shoolini University, Solan, India; 230 University of Hyderabad, Hyderabad, India; 231 University of Visva-Bharati, Santiniketan, India; 232 Indian Institute of Technology (IIT), Mumbai, India; 233 Deutsches Elektronen-Synchrotron, Hamburg, Germany; 234 Department of Physics, University of Science and Technology of Mazandaran, Behshahr, Iran; 235 INFN Sezione di Baria, Università di Barib, Politecnico di Bari, Bari, Italy; 236 Italian National Agency for New Technologies, Energy and Sustainable Economic Development, Bologna, Italy; 237 Centro Siciliano di Fisica Nucleare e di Struttura Della Materia, Catania, Italy; 238 Riga Technical University, Riga, Latvia, Riga, Latvia; 239 Consejo Nacional de Ciencia y Tecnología, Mexico City, Mexico; 240 Warsaw University of Technology, Institute of Electronic Systems, Warsaw, Poland; 241 Institute for Nuclear Research, Moscow, Russia; 242 National Research Nuclear University ’Moscow Engineering Physics Institute’ (MEPhI), Moscow, Russia; 243 Institute of Nuclear Physics of the Uzbekistan Academy of Sciences, Tashkent, Uzbekistan; 244 St. Petersburg State Polytechnical University, St. Petersburg, Russia; 245 University of Florida, Gainesville, USA; 246 Imperial College, London, UK; 247 P.N. Lebedev Physical Institute, Moscow, Russia; 248 California Institute of Technology, Pasadena, USA; 249 Budker Institute of Nuclear Physics, Novosibirsk, Russia; 250 Faculty of Physics, University of Belgrade, Belgrade, Serbia; 251 Università degli Studi di Siena, Siena, Italy; 252 Trincomalee Campus, Eastern University, Sri Lanka, Nilaveli, Sri Lanka; 253 INFN Sezione di Pavia, Università di Pavia, Pavia, Italy; 254 National and Kapodistrian University of Athens, Athens, Greece; 255 Universität Zürich, Zurich, Switzerland; 256 Stefan Meyer Institute for Subatomic Physics, Vienna; Austria, Vienna, Austria; 257 Laboratoire d’Annecy-le-Vieux de Physique des Particules, IN2P3-CNRS, Annecy-le-Vieux, France; 258 Şırnak University, Sirnak, Turkey; 259 Department of Physics, Tsinghua University, Beijing; China, Beijing, China; 260 Near East University, Research Center of Experimental Health Science, Nicosia, Turkey; 261 Beykent University, Istanbul; Turkey, Istanbul, Turkey; 262 Istanbul Aydin University, Application and Research Center for Advanced Studies (App. & Res. Cent. for Advanced Studies), Istanbul, Turkey; 263 Mersin University, Mersin, Turkey; 264 Piri Reis University, Istanbul, Turkey; 265 Adiyaman University, Adiyaman, Turkey; 266 Ozyegin University, Istanbul, Turkey; 267 Izmir Institute of Technology, Izmir, Turkey; 268 Necmettin Erbakan University, Konya, Turkey; 269 Bozok Universitetesi Rektörlügü, Yozgat; Turkey, Yozgat, Turkey; 270 Marmara University, Istanbul, Turkey; 271 Milli Savunma University, Istanbul, Turkey; 272 Kafkas University, Kars, Turkey; 273 Istanbul Bilgi University, Istanbul, Turkey; 274 Hacettepe University, Ankara, Turkey; 275 School of Physics and Astronomy, University of Southampton, Southampton, UK; 276 IPPP Durham University, Durham, UK; 277 Monash University, Faculty of Science, Clayton, Australia; 278 Bethel University, St. Paul, Minneapolis; USA, St. Paul, USA; 279 Karamanoğlu Mehmetbey University, Karaman, Turkey; 280 Ain Shams University, Cairo, Egypt; 281 Bingol University, Bingol, Turkey; 282 Georgian Technical University, Tbilisi, Georgia; 283 Sinop University, Sinop, Turkey; 284 Mimar Sinan University, Istanbul, Istanbul, Turkey; 285 Nanjing Normal University Department of Physics, Nanjing, China; 286 Texas A&M University at Qatar, Doha, Qatar; 287 Kyungpook National University, Daegu, Korea, Daegu, Korea; 288 CERN, 1211 Geneva 23, Switzerland

## Abstract

This paper presents new sets of parameters (“tunes”) for the underlying-event model of the ${\textsc {herwig}} \,7$ event generator. These parameters control the description of multiple-parton interactions (MPI) and colour reconnection in ${\textsc {herwig}} \,7$, and are obtained from a fit to minimum-bias data collected by the CMS experiment at $\sqrt{s}=0.9$, 7, and $13 \,\text {Te}\text {V}$. The tunes are based on the NNPDF 3.1 next-to-next-to-leading-order parton distribution function (PDF) set for the parton shower, and either a leading-order or next-to-next-to-leading-order PDF set for the simulation of MPI and the beam remnants. Predictions utilizing the tunes are produced for event shape observables in electron-positron collisions, and for minimum-bias, inclusive jet, top quark pair, and Z and W boson events in proton-proton collisions, and are compared with data. Each of the new tunes describes the data at a reasonable level, and the tunes using a leading-order PDF for the simulation of MPI provide the best description of the data.

## Introduction

In hadron-hadron collisions, the hard scattering of partons is accompanied by additional activity from multiple-parton interactions (MPI) that take place within the same collision, and by interactions between the remnants of the hadrons. To describe the underlying-event (UE) activity in a hard scattering process, and minimum-bias (MB) events, Monte Carlo (MC) event generators such as ${\textsc {herwig}} \,7$ [1–3] and ${\textsc {pythia}} \,8$ [4] include a model of these additional interactions. Because these processes are soft in nature, perturbative quantum chromodynamics (QCD) cannot be used to predict them, so they must be described by a phenomenological model. The parameters of the models must be optimized to provide a reasonable description of measured observables that are sensitive to the UE and MB events. An accurate description of the UE by MC event generators, along with an understanding of the uncertainties in the description, is of particular importance for precision measurements at hadron colliders, such as the extraction of the top quark mass. This paper presents new sets of parameters (“tunes”) for the UE model of the ${\textsc {herwig}} \,7$ event generator.

The ${\textsc {herwig}} \,7$ event generator is a multipurpose event generator, which can perform matrix-element (ME) calculations beyond leading order (LO) in QCD, via the matchbox module [5], matched with parton shower (PS) calculations. Both an angular-ordered and a dipole-based PS simulation are available in ${\textsc {herwig}} \,7$, and the former is used in this paper. The ME calculations can also be provided by an external ME generator, such as powheg [6–8] or MadGraph 5_amc@nlo [9]. The ${\textsc {herwig}} \,7$ generator is built upon the development of the preceding herwig [10] and herwig++ [1] event generators. In addition to the simulation of hard scattering of partons in hadron-hadron collisions, a simulation of MPI, which is modelled by a combination of soft and hard interactions and by colour reconnection (CR) [1, 11–13], is included in ${\textsc {herwig}} \,7$. As shown in Ref. [13], a model of CR is required in ${\textsc {herwig}} \,7$ to describe the structure of colour connections between different MPI, and to obtain a good description of the mean charged-particle transverse momentum ($p_{\mathrm {T}}$) as a function of the charged-particle multiplicity ($N_{\text {ch}}$).

The model describing the soft interactions, and also diffractive processes, was improved in version 7.1 of ${\textsc {herwig}} \,7$. This resulted in a new tune of the MPI parameters, called $\text {SoftTune}$, which improved the description of MB data [3, 12]. In particular, the description of final-state hadronic systems separated by a large rapidity gap [14, 15] is notably improved because a significant contribution is expected from diffractive events. The tune $\text {SoftTune}$ is based on the MMHT 2014 LO parton distribution function (PDF) set [16], and was derived by fitting MB data at $\sqrt{s} =0.9,~7,$ and $13\,\text {Te}\text {V}$ from the ATLAS experiment [17]. The MB data used in the tuning include the pseudorapidity ($\eta$) and $p_{\mathrm {T}}$ distributions of charged particles for various lower bounds on $N_{\text {ch}}$, namely $N_{\text {ch}} \ge 1,~2,~6,$ and 20. The mean charged-particle $p_{\mathrm {T}}$ as a function of $N_{\text {ch}}$ was also included in the tuning procedure. Three models of CR are available in ${\textsc {herwig}} \,7$, and $\text {SoftTune}$ was derived with the plain colour reconnection (PCR) model implemented. The same PCR model is considered in our studies.

In this paper, we present new UE tunes for the ${\textsc {herwig}} \,7$ (version 7.1.4) generator. In contrast to $\text {SoftTune}$, the tunes presented here are based on the NNPDF 3.1 PDF sets [18], and use the next-to-next-to-leading-order (NNLO) PDF set for the simulation of the PS, and either an LO or NNLO PDF set for the simulation of MPI and the beam remnants. This choice of PDF sets is similar to that used to obtain tunes for the ${\textsc {pythia}} \,8$ event generator in Ref. [19], where it was shown that predictions from ${\textsc {pythia}} \,8$ using LO, next-to-leading-order (NLO), and NNLO PDFs with their associated tunes can all give a reliable description of the UE. Based on these findings and the wide use by the CMS Collaboration of the CP5 ${\textsc {pythia}} \,8$ tune, we concentrate on deriving tunes for the ${\textsc {herwig}} \,7$ generator that are also based on an NNLO PDF set for the simulation of the parton shower. It is verified that using an NNLO PDF in the simulation of the PS in ${\textsc {herwig}} \,7$ also provides a reliable description of MB data. A consistent choice of PDF in the ${\textsc {herwig}} \,7$ and ${\textsc {pythia}} \,8$ generators, as well as a similar method of the MPI model tuning, provides a better comparison of predictions from these two generators.

The tunes are derived by fitting measurements from proton-proton collision data collected by the CMS experiment [20] at $\sqrt{s} = 0.9$, 7, and $13\,\text {Te}\text {V}$. The measurements used in the fitting procedure are chosen because of their sensitivity to the modelling of the UE in ${\textsc {herwig}} \,7$. Uncertainties in the parameters of one of the new tunes are also derived. This quantifies the effect of the uncertainties in the fitted parameters for future analyses. To validate the performance of the new tunes, the corresponding ${\textsc {herwig}} \,7$ predictions are compared with a range of MB data from proton-proton and proton-antiproton collisions. Comparisons are also made using event shape observables from electron-positron collisions collected at the CERN LEP accelerator, which are particularly sensitive to the choice of the strong coupling $\alpha _\mathrm {S}$ in the description of final-state radiation. To further validate the new tunes, predictions of differential $\mathrm{t}{\bar{\mathrm{t}}}$ , Z boson, and W boson cross sections are also obtained from matching ME calculations from powheg and MadGraph 5_amc@nlo with the ${\textsc {herwig}} \,7$ PS description. The kinematics of the $\mathrm{t}{\bar{\mathrm{t}}}$ system are studied, along with the multiplicity of additional jets, which are sensitive to the modelling by the PS simulation, in $\mathrm{t}{\bar{\mathrm{t}}}$ , Z boson, and W boson events. The modelling of the UE in Z boson events, and the substructure of jets in $\mathrm{t}{\bar{\mathrm{t}}}$ and in inclusive jet events are also investigated. Some of these comparisons are sensitive to the modelling by the ME calculations, and the purpose of those is to validate that the various predictions using the tunes do not differ from each other by a significant amount. Other comparisons are more sensitive to the modelling of the PS and MPI simulation, allowing us to test the new tunes in data other than MB data.

This paper is organized as follows. In Sect. 2, we summarize the UE model employed by ${\textsc {herwig}} \,7$, and describe the model parameters considered in the tuning. The choice of PDF and the value of the strong coupling in the tunes is discussed in Sect. 3 in addition to details of the fitting procedure. The new tunes are presented in Sect. 4, and the corresponding predictions from ${\textsc {herwig}} \,7$ are compared with MB data. Uncertainties in one of the derived tunes are presented in Sect. 5. Further validation of the new tunes is performed in the following sections: their predictions are compared with event shape observables from the CERN LEP in Sect. 6, and with top quark, inclusive jet, and Z and W boson production data in Sects. 7, 8, and 9, respectively. Finally, we present a summary in Sect. 10.

**Table 1:** Parameters considered in the tuning, and their allowed ranges in the fit

Parameter	${\textsc {herwig}} \,7$ configuration parameter	Range
$p_{\bot ,\mathrm {0}}^\mathrm {min}$ ($\text {Ge}\text {V}$)	/Herwig/UnderlyingEvent/MPIHandler:pTmin0	1.0–5.0
$b$	/Herwig/UnderlyingEvent/MPIHandler:Power	0.1–0.5
$\mu ^{2}$ ($\text {Ge}\text {V} ^{-2}$)	/Herwig/UnderlyingEvent/MPIHandler:InvRadius	0.5–2.7
$p_{\text {reco}}$	/Herwig/Hadronization/ColourReconnector:ReconnectionProbability	0.05–0.90

## The UE model in HERWIG 7

The UE in ${\textsc {herwig}} \,7$ is modelled by a combination of soft and hard interactions [1, 11, 12]. The parameter $p_{\bot }^\text {min}$ defines the transition between the soft and hard MPI. The interactions with a pair of outgoing partons with $p_{\mathrm {T}}$ above $p_{\bot }^\text {min}$ are treated as hard interactions, which are constructed from QCD two-to-two processes. The $p_{\bot }^\text {min}$ transition threshold depends on the centre-of-mass energy of the hadron-hadron collision and is given by:

1 $$\begin{aligned} p_{\bot }^\text {min} = p_{\bot ,\mathrm {0}}^\mathrm {min} \left( \frac{\sqrt{s}}{E_{\mathrm {0}}}\right) ^b, \end{aligned}$$

where $p_{\bot ,\mathrm {0}}^\mathrm {min}$ is the value of $p_{\bot }^\text {min}$ at a reference energy scale $E_{\mathrm {0}}$, which is set to 7$\,\text {Te}\text {V}$, $\sqrt{s}$ is the centre-of-mass energy of the hadron-hadron collision, and the parameter $b$ controls the energy dependence of $p_{\bot }^\text {min}$. Decreasing the value of $p_{\bot }^\text {min}$ increases the number of hard interactions whilst reducing the number of soft interactions, which typically increases the amount of activity in the UE.

The average number $\langle n\rangle$ of these additional hard interactions per hadron-hadron collision is given by:

2 $$\begin{aligned} \langle n\rangle =A(d) \sigma (s), \end{aligned}$$

where $\sigma (s)$ is the production cross section of a pair of partons with $p_{\mathrm {T}} >p_{\bot }^\text {min}$ and $A(d)$ describes the overlap between the two protons at a given impact parameter $d$. The form of the overlap function is given by:

3 $$\begin{aligned} A(d) = \frac{\mu ^{2}}{96\pi }(\mu d)^{3} K_{3}, \end{aligned}$$

where $$\mu ^{2} $$ is the inverse proton radius squared, and $$K_{3}\equiv {K_{3}(\mu d)}$$ is the modified Bessel function of the third kind. The overlap function is obtained by the convolution of the electromagnetic form factors of two protons. The number of additional hard interactions per hadron-hadron collision at a given $$d $$ is described by a Poissonian probability distribution with a mean given by Eq. (2), which is then integrated over the impact parameter space. Increasing $$\mu ^{2}$$ increases the density of the partons in the hadrons, and results in a higher probability for additional hard scatterings to take place.

Additional soft interactions, which produce pairs of partons below $p_{\bot }^\text {min}$, are based on a model of multiperipheral particle production [12]. The number of additional soft interactions between the two hadron remnants is described in a similar way to the hard interactions above $p_{\bot }^\text {min}$. In a soft interaction between the two hadron remnants, the mean number of particles produced is given by:

4 $$\begin{aligned} \langle N \rangle = N_\mathrm {0} \left( \frac{s}{1 \,\text {Te}\text {V} ^2} \right) ^P \ln \frac{\left( p_{\mathrm {r1}} + p_{\mathrm {r2}}\right) ^2}{m^{2}_{\mathrm {rem}}}, \end{aligned}$$

where $p_{\mathrm {r1}}$ and $p_{\mathrm {r2}}$ are the four-momenta of the two remnants, and $m_{\mathrm {rem}}$ is the mass of a proton remnant, i.e. the remaining valence quarks of a proton treated as a diquark system, and is set to $0.95\,\text {Ge}\text {V}$. The parameters $N_\mathrm {0}$ and $P$ control the energy dependence of the mean number of soft particles produced. They were tuned to MB data, which resulted in the values $P =-0.08$ and $N_\mathrm {0} =0.95$ [3]. In deriving the tune $\text {SoftTune}$ the values of $N_\mathrm {0}$ and $P$ were kept fixed at these values.

The cluster model [21] is used to model the hadronization of quarks into hadrons. After the PS calculation, gluons are split into quark-antiquark pairs, and a cluster is formed from each colour connected pair of quarks. Before hadrons are produced from the clusters, CR can modify the configuration of the clusters. With the PCR model, the quarks from two clusters can be reconfigured to form two alternative clusters. The change of the cluster configuration takes place only if the sum of the masses of the new clusters is smaller than before. If this condition is satisfied, the CR is accepted with a probability $p_{\text {reco}}$, which is the only parameter of the PCR model. The PCR model typically leads to clusters with smaller invariant mass compared with the clusters that would be obtained without CR, and will typically reduce the overall activity in the UE.

## Tuning procedure

We derive three tunes based on the NNPDF 3.1 PDF sets [18]. A different PDF set is chosen for each aspect of the ${\textsc {herwig}} \,7$ simulation: hard scattering, parton showering, MPI, and beam remnant handling. The value of $\alpha _\mathrm {S}$ at a scale equal to the Z boson mass $m_{\mathrm {\mathrm{Z}\,}}$in each tune is set to $\alpha _\mathrm {S} (m_{\mathrm {\mathrm{Z}\,}})=0.118$ for all parts of the ${\textsc {herwig}} \,7$ simulation, with a two-loop running of $\alpha _\mathrm {S}$.

**Fig. 1:**
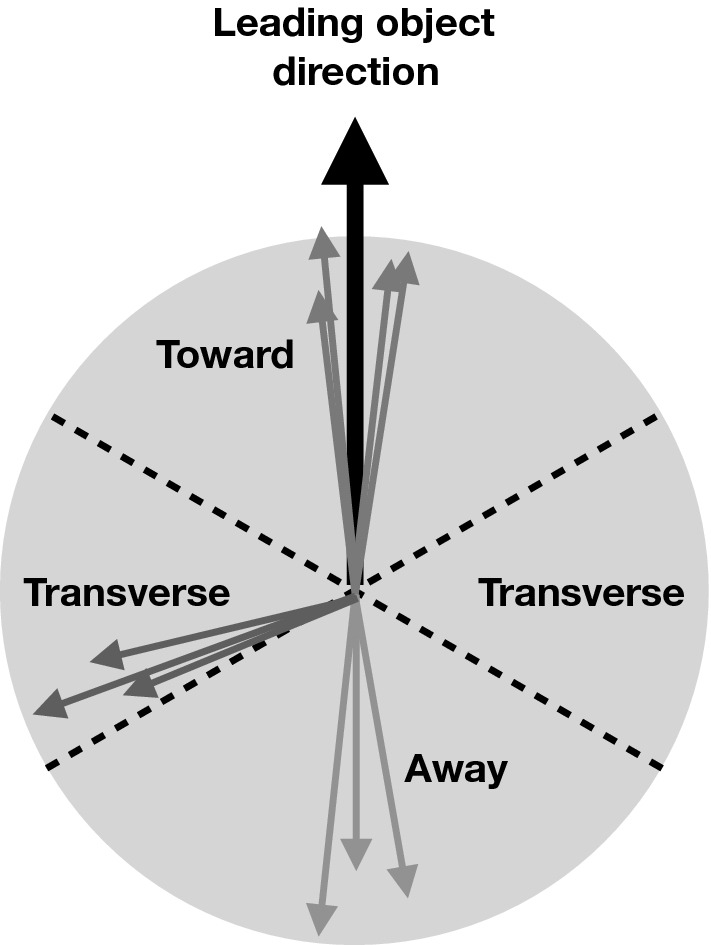
Illustration of the different $\phi$ regions, with respect to the leading object in an event, used to probe the properties of the UE in measurements

**Table 2:** Value of the parameters for the $\text {SoftTune}$ [3, 12], CH1, CH2, and CH3 tunes

		$\text {SoftTune}$	CH1	CH2	CH3
$\alpha _\mathrm {S} (m_{\mathrm {\mathrm{Z}\,}})$		0.1262	0.118	0.118	0.118
PS	PDF set	MMHT 2014 LO	NNPDF 3.1 NNLO	NNPDF 3.1 NNLO	NNPDF 3.1 NNLO
	$\alpha _\mathrm {S} ^{\mathrm {PDF}}(m_{\mathrm {\mathrm{Z}\,}})$	0.135	0.118	0.118	0.118
MPI&	PDF set	MMHT 2014 LO	NNPDF 3.1 NNLO	NNPDF 3.1 LO	NNPDF 3.1 LO
remnants	$\alpha _\mathrm {S} ^{\mathrm {PDF}}(m_{\mathrm {\mathrm{Z}\,}})$	0.135	0.118	0.118	0.130
$p_{\bot ,\mathrm {0}}^\mathrm {min}$ ($\text {Ge}\text {V}$)		3.502	2.322	3.138	3.040
$b$		0.416	0.157	0.120	0.136
$\mu ^{2}$ ($\text {Ge}\text {V} ^{-2}$)		1.402	1.532	1.174	1.284
$p_{\text {reco}}$		0.5	0.400	0.479	0.471
$\chi ^{2}$/$N_{\text {dof}}$		12.8	6.75	1.54	1.71

The first tune, CH1 (“CMS herwig ”), uses an NNLO PDF set in all aspects of simulation in ${\textsc {herwig}} \,7$, where the PDF was derived with a value of $\alpha _\mathrm {S} (m_{\mathrm {\mathrm{Z}\,}}) =0.118$. This is equivalent to the choice of PDF and $\alpha _\mathrm {S} (m_{\mathrm {\mathrm{Z}\,}})$ used in the CP5 ${\textsc {pythia}} \,8$ tune [19]. In the second tune, CH2, an LO PDF set that was also derived with $\alpha _\mathrm {S} (m_{\mathrm {\mathrm{Z}\,}}) =0.118$, is used in the simulation of MPI and beam remnant handling, whereas an NNLO PDF set is used elsewhere. The final tune, CH3, is similar to CH2, but uses an LO PDF set that was derived with $\alpha _\mathrm {S} (m_{\mathrm {\mathrm{Z}\,}}) =0.130$ for the simulation of MPI and remnant handling. The choice of an LO PDF set for the simulation of MPI and beam remnant handling, regardless of the choice of PDF used in the PS and ME calculation, is motivated by ensuring that the gluon PDF is positive at the low energy scales involved, which is not necessarily the case with higher-order PDF sets. However, as was shown in Ref. [19], the gluon PDF in the NNLO NNPDF 3.1 set remains positive at low energy scales, and predictions from ${\textsc {pythia}} \,8$ using LO and higher-order PDFs can both give a reliable description of MB data. The configurations of PDF sets in the CH1, CH2, and CH3 tunes allow us to study whether using an NNLO PDF set consistently for all aspects of the ${\textsc {herwig}} \,7$ simulation, or an LO PDF set for the simulation of MPI, can both give a reliable description of MB data. For both of these choices the gluon PDF is positive at low energy scales.

The names of the parameters being tuned in the ${\textsc {herwig}} \,7$ configuration, and their allowed ranges in the fit, are shown in Table 1. The values of $N_\mathrm {0} =0.95$ and $P =-0.08$ are fixed at the values that were used in the tune $\text {SoftTune}$. As shown later, no further tuning of these parameters is necessary, because of the good description of measured observables obtained with these values.

The tunes are derived by fitting unfolded MB data that are available in the rivet [22] toolkit. The proton-proton collision data used in the fit were collected by the CMS experiment at $\sqrt{s} =0.9$, 7, and 13$\,\text {Te}\text {V}$. In measurements probing the UE, charged particles in a particular event are typically categorized into different $\eta$-$\phi$ regions with respect to a leading object in that event, such as the highest $p_{\mathrm {T}}$ track or jet, as illustrated in Fig. 1. The difference in azimuthal $\phi$ between each charged particle and the leading object ($\varDelta \phi$) is used to assign each charged particle to a region, namely the toward ($|\varDelta \phi |\le 60^{\circ }$), away ($|\varDelta \phi |>120^{\circ }$), and transverse regions ($60<|\varDelta \phi |\le 120^{\circ }$). The properties of the charged particles in the transverse regions are the most sensitive to the modelling of the UE. The two transverse regions can be further divided into the transMin and transMax regions, which are the regions with the least and most charged-particle activity, respectively. Data that have been categorized in this way are referred to as UE data in this paper.

**Fig. 2:**
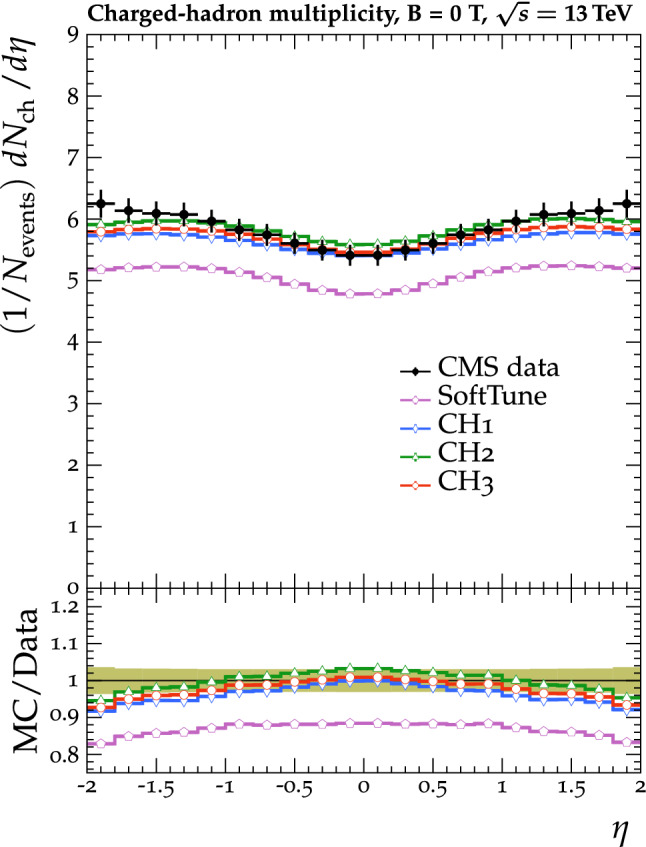
The normalized $\mathrm {d} N_{\text {ch}}/\mathrm {d} \eta$ of charged hadrons as a function of $\eta$ [27]. CMS MB data are compared with $\text {SoftTune}$ and the CH tunes. The coloured band in the ratio plot represents the total experimental uncertainty in the data. The vertical bars on the points for the different predictions represent the statistical uncertainties

**Fig. 3:**
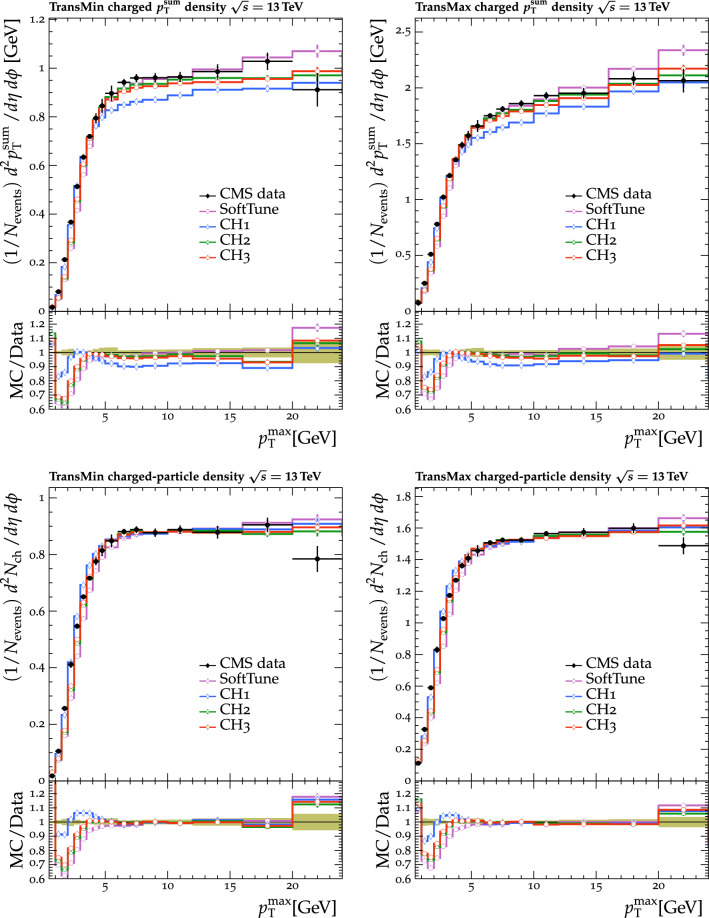
The normalized $p_{\mathrm {T}} ^{\text {sum}}$ (upper) and $N_{\text {ch}}$ (lower) density distributions in the transMin (left) and transMax (right) regions, as a function of the $p_{\mathrm {T}}$ of the leading track, $p_{\mathrm {T}} ^{\text {max}}$ [24]. CMS MB data are compared with the predictions from ${\textsc {herwig}} \,7$, with the $\text {SoftTune}$ and CH tunes. The coloured band in the ratio plot represents the total experimental uncertainty in the data. The vertical bars on the points for the different predictions represent the statistical uncertainties

**Fig. 4:**
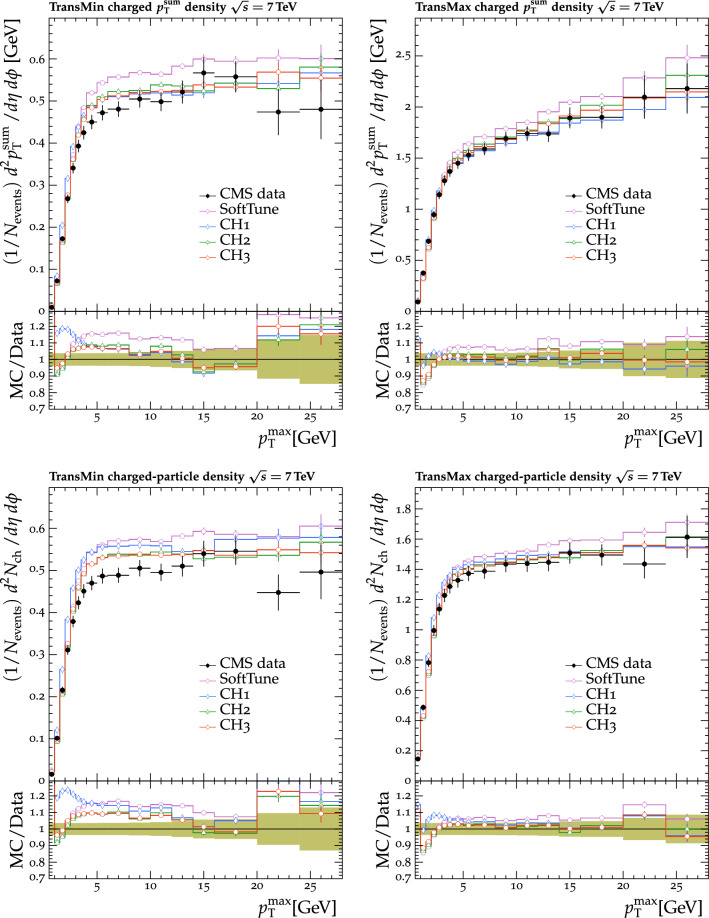
The $p_{\mathrm {T}} ^{\text {sum}}$ (upper) and $N_{\text {ch}}$ (lower) density distributions in the transMin (left) and transMax (right) regions, as a function of the $p_{\mathrm {T}}$ of the leading track, $p_{\mathrm {T}} ^{\text {max}}$ [23]. CMS MB data are compared with the predictions from ${\textsc {herwig}} \,7$, with the $\text {SoftTune}$ and CH tunes. The coloured band in the ratio plot represents the total experimental uncertainty in the data. The vertical bars on the points for the different predictions represent the statistical uncertainties

**Fig. 5:**
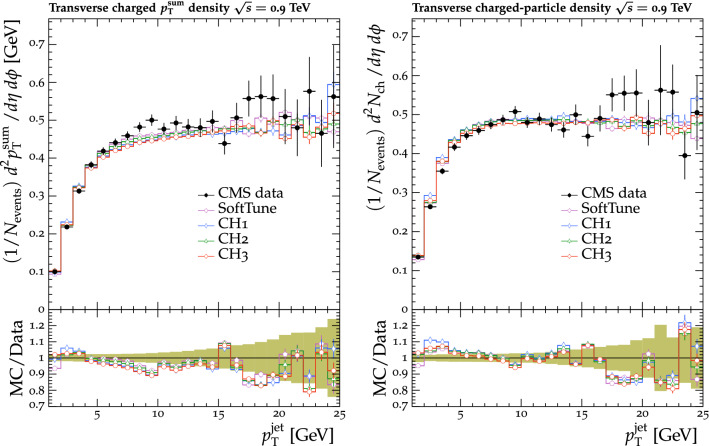
The $p_{\mathrm {T}} ^{\text {sum}}$ (left) and $N_{\text {ch}}$ (right) density distributions in the transverse regions, as a function of the $p_{\mathrm {T}}$ of the leading track jet, $p_{\mathrm {T}} ^{\text {jet}}$ [25]. CMS MB data are compared with the predictions from ${\textsc {herwig}} \,7$, with the $\text {SoftTune}$ and CH tunes. The coloured band in the ratio plot represents the total experimental uncertainty in the data. The vertical bars on the points for the different predictions represent the statistical uncertainties

**Fig. 6:**
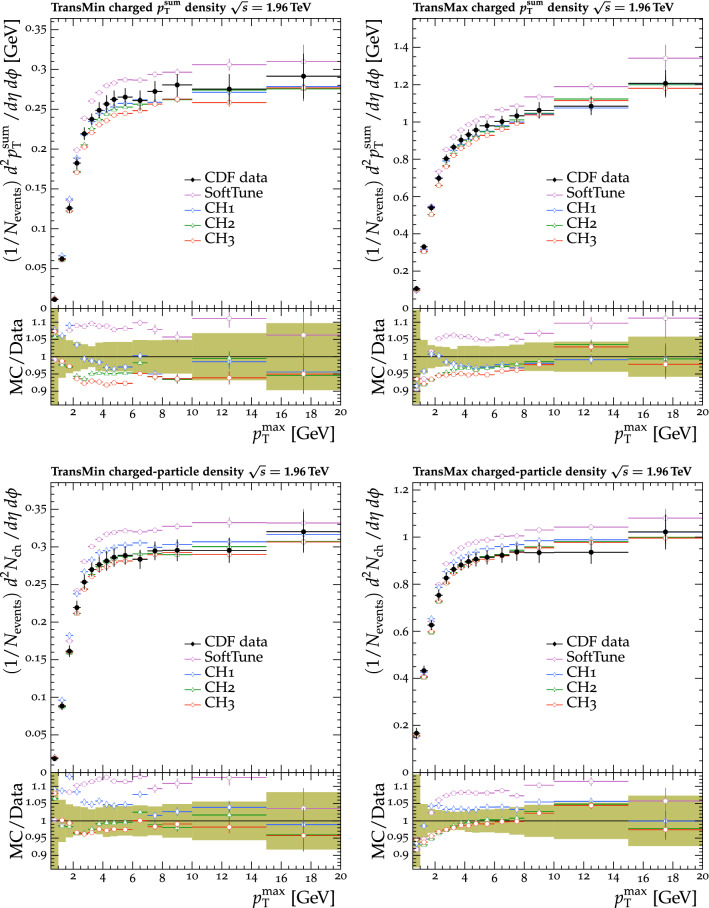
The $p_{\mathrm {T}} ^{\text {sum}}$ (upper) and $N_{\text {ch}}$ (lower) density distributions in the transMin (left) and transMax (right) regions, as a function of the $p_{\mathrm {T}}$ of the leading track, $p_{\mathrm {T}} ^{\text {max}}$ [31]. CDF MB data are compared with the predictions from ${\textsc {herwig}} \,7$, with the $\text {SoftTune}$ and CH tunes. The coloured band in the ratio plot represents the total experimental uncertainty in the data. The vertical bars on the points for the different predictions represent the statistical uncertainties

**Fig. 7:**
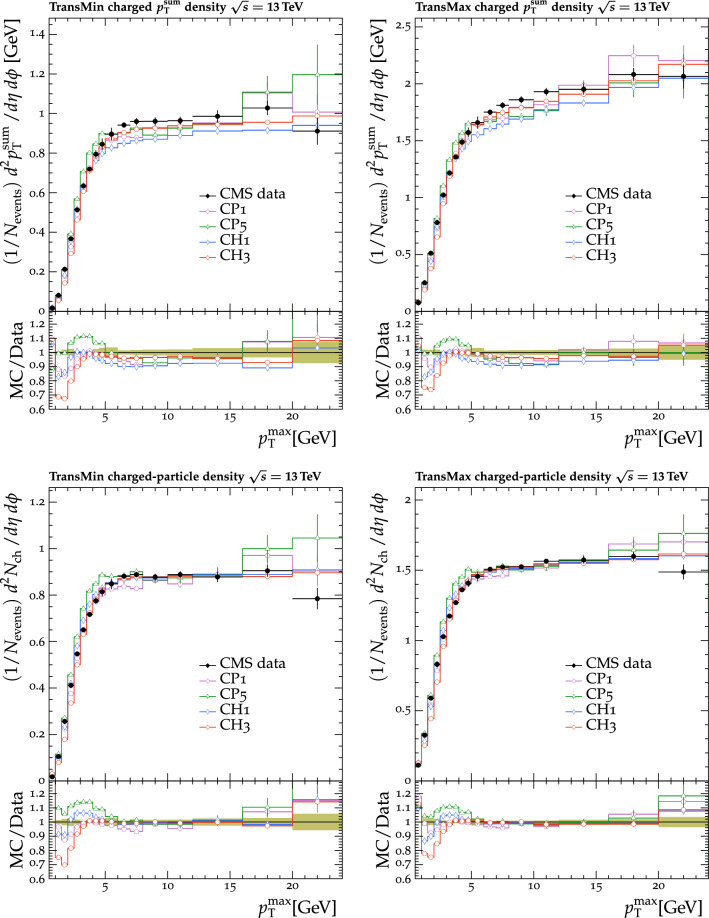
The $p_{\mathrm {T}} ^{\text {sum}}$ (upper) and $N_{\text {ch}}$ (lower) density distributions in the transMin (left) and transMax (right) regions, as a function of the $p_{\mathrm {T}}$ of the leading track, $p_{\mathrm {T}} ^{\text {max}}$ [24]. CMS MB data are compared with the predictions from ${\textsc {herwig}} \,7$, with the CH1 and CH3 tunes, and from ${\textsc {pythia}} \,8$, with the CP1 and CP5 tunes. The coloured band in the ratio plot represents the total experimental uncertainty in the data. The vertical bars on the points for the different predictions represent the statistical uncertainties

At $\sqrt{s} =7$ and 13$\,\text {Te}\text {V}$, the $N_{\text {ch}}$ and transverse momentum sum ($p_{\mathrm {T}} ^{\text {sum}}$), with respect to the beam axis, as functions of the $p_{\mathrm {T}}$ of the leading track ($p_{\mathrm {T}} ^{\text {max}}$) in the transMin and transMax regions are used in the fit [23, 24]. At $\sqrt{s} =0.9\,\text {Te}\text {V}$, the observables used are the $N_{\text {ch}}$ and $p_{\mathrm {T}} ^{\text {sum}}$ in the transverse region, as a function of the $p_{\mathrm {T}}$ of the leading jet ($p_{\mathrm {T}} ^{\text {jet}}$) [25]. The track jets are clustered using the SISCone algorithm [26] with a distance parameter of 0.5. The regions $p_{\mathrm {T}} ^{\text {max}} <3\,\text {Ge}\text {V}$ and $p_{\mathrm {T}} ^{\text {jet}} <3\,\text {Ge}\text {V}$ are not included in the fit because the parameters of diffractive processes, which dominate this region, are not considered. The charged-hadron multiplicity as a function of $\eta$, $\mathrm {d} N_{\text {ch}}/\mathrm {d} \eta$, as measured by CMS at $\sqrt{s} =13\,\text {Te}\text {V}$ with zero magnetic field strength ($\mathrm {B}=0\,\text {T}$) [27] is also used in the fitting procedure. The charged-particle $p_{\mathrm {T}}$ and $\eta$ as measured by CMS in Ref. [28] are not considered here, since they are biased by predictions obtained with ${\textsc {pythia}} \,6$ [29], as discussed in Ref. [12].

The tuning is performed within the professor (v1.4.0) framework [30]. Around 60 random choices of the parameters are made, and predictions for each of these choices are obtained using rivet. Approximately 10 million MB events are generated for each choice of parameters, such that the uncertainty in the prediction in any bin is typically not larger than the uncertainty in the data in the same bin.

The fit is performed by minimising the $\chi ^{2}$ function:

5 $$\begin{aligned} \chi ^{2} (p) = \sum _{{\mathcal {O}}}w_{{\mathcal {O}}} \sum _{i \in {\mathcal {O}}}\frac{(f^{i}(p) -{\mathcal {R}}_i)^2}{\varDelta ^2_i}, \end{aligned}$$

where ${\mathcal {R}}_i$ is the measured content of bin $i$ of the distribution of observable ${\mathcal {O}}$, while $f^{i}(p)$ is the predicted content in bin $i$, which is obtained by professor from a parameterization of the dependence of the prediction on the tuning parameters *p*. The total uncertainty in the data and the simulated prediction in bin $i$ of a given observable is denoted by $\varDelta ^2_i$, and $w_{{\mathcal {O}}}$ is a weight that increases or decreases the importance of an observable ${\mathcal {O}}$ in the fit. The weight is typically set to $w_{{\mathcal {O}}} =1$. However, for the CH1 tune, where the PDF set used in the simulation of MPI and beam remnants is an NNLO set instead of an LO set, the weight is set to $w_{{\mathcal {O}}} =3$ for the $\mathrm {d} N_{\text {ch}}/\mathrm {d} \eta$ distribution. This is the smallest weight that ensures the distribution is well described after the tuning. Beyond this, the parameters for the three tunes and their predictions are stable with respect to a change in the weight assigned to the $\mathrm {d} N_{\text {ch}}/\mathrm {d} \eta$ distribution in the fit. Correlations between the bins $i$ are not taken into account when minimising Eq. (5), because these were not available for the used input distributions. A third-order polynomial is used to parameterize the dependence of the prediction on the tuning parameters. Using a fourth-order polynomial to perform this interpolation between the 60 choices of parameters has a negligible effect on the outcome of the fits.

The number of degrees of freedom ($N_{\text {dof}}$) in the fit is calculated as:

6 $$\begin{aligned} N_{\text {dof}} = \frac{(\sum _{{\mathcal {O}}}\sum _{i \in {\mathcal {O}}}w_{{\mathcal {O}}})^2}{\sum _{{\mathcal {O}}} \sum _{i \in {\mathcal {O}}}w_{{\mathcal {O}}} ^2} - N_{\text {param}}, \end{aligned}$$

where $N_{\text {param}}$ is the number of parameters being optimized in the fit.

## Results from the new HERWIG 7 tunes

The tuned values of the parameters and the $\chi ^{2}$ values from the fit, i.e. the minimum values of Eq. (5), divided by the $N_{\text {dof}}$ of the fit are shown in Table 2, along with the values of the parameters for the default tune $\text {SoftTune}$. The $N_{\text {dof}}$ in the fit is 118 for CH1, and 152 for CH2 and CH3. To provide a comparison between the compatibilities of the CH tunes and $\text {SoftTune}$ with the data, the $\chi ^{2}$/$N_{\text {dof}}$ corresponding to the prediction of SoftTune and the data is also shown with $N_{\text {dof}}$ set to 152.

The values of the parameters of the MPI model are intertwined with each other since they are tuned simultaneously to reproduce the amount of UE activity observed in the data. Nonetheless, a general interpretation of the variations in the tuned parameters for each tune can be distinguished. For example, the value of $p_{\bot ,\mathrm {0}}^\mathrm {min}$ is lower for all three CH tunes than for $\text {SoftTune}$, and significantly lower for CH1, which increases the amount of MPI in an event compared to that with the tune $\text {SoftTune}$.

The lower value of $b$ for all CH tunes further increases the contribution of MPI in collisions at $\sqrt{s} =13\,\text {Te}\text {V}$. Because of the lower values of $p_{\text {reco}}$, the amount of CR in the CH tunes is lower than in $\text {SoftTune}$. This also has the effect of increasing the overall amount of activity in the UE for the CH tunes. The value of $\mu ^{2}$ for CH2 and CH3 is lower than the corresponding value for $\text {SoftTune}$. Even though a lower value of $\mu ^{2}$ would lead to a lower amount of MPI in a given event, the combined effect of the parameters of the CH tunes results in a larger amount of MPI compared with $\text {SoftTune}$.

The tuned parameters of CH2 and CH3 are fairly similar, as are the values of $\chi ^{2}$/$N_{\text {dof}}$ of these two tunes, indicating that the choice of $\alpha _\mathrm {S} (m_{\mathrm {\mathrm{Z}\,}})$ used when deriving the LO PDF set in the simulation of MPI does not have a large effect. The parameters for the tune CH1 differ from those for the tunes CH2 and CH3, and the value of $\chi ^{2}$/$N_{\text {dof}}$ is larger, implying that using an LO PDF set is somewhat preferred over an NNLO PDF set for the simulation of MPI. In the following, the predictions from the three CH tunes are compared with the data used in the tuning procedure. These predictions are obtained by generating events with the corresponding parameters shown in Table 2 rather than from the parameterization of the tune parameters used in the fit.

Figure 2 shows the normalized $\mathrm {d} N_{\text {ch}}/\mathrm {d} \eta$ of charged hadrons as a function of $\eta$ at 13$\,\text {Te}\text {V}$ in MB events. Only the predictions for $\text {SoftTune}$ deviate significantly from the data, and underestimate the $\mathrm {d} N_{\text {ch}}/\mathrm {d} \eta$ in data by 10–18%. The CH tunes each provide a slightly different prediction, but all have a similar level of agreement with the data. The CH tunes compared with SoftTune predict an increase in the UE activity, which is observed.

Figure 3 shows the normalized $p_{\mathrm {T}} ^{\text {sum}}$ and $N_{\text {ch}}$ densities as a function of $p_{\mathrm {T}} ^{\text {max}}$ with comparisons from $\text {SoftTune}$ and the CH tunes for both transMin and transMax. The predictions of $\text {SoftTune}$ and the CH2, CH3 tunes are broadly similar, and give a good description the data in the plateau region ($p_{\mathrm {T}} ^{\text {max}} \gtrsim 4\,\text {Ge}\text {V}$). In the rising part of the spectrum, the predictions from the tunes CH2, CH3, and $\text {SoftTune}$ deviate from the data in some bins by up to 40%. The CH3 tune provides the best predictions in the rising region of the spectrum. However, only the region $p_{\mathrm {T}} ^{\text {max}} >3\,\text {Ge}\text {V}$ was included in the tuning procedure, because the region $p_{\mathrm {T}} ^{\text {max}} <3\,\text {Ge}\text {V}$ is dominated by diffractive processes whose model parameters are not used in the fit.

The effect of using an NNLO PDF, instead of an LO PDF, in the simulation of MPI is seen from the predictions with the tune CH1 in Fig. 3. This tune provides a good description of the $N_{\text {ch}}$ distributions in both the transMin and transMax regions, and is typically within 10% of the data. However, the tune CH1 does not simultaneously provide a good description of the $p_{\mathrm {T}} ^{\text {sum}}$ distributions in either the transMin or transMax region, with a 10% difference to the data in the plateau region of the corresponding transMax distribution.

**Fig. 8:**
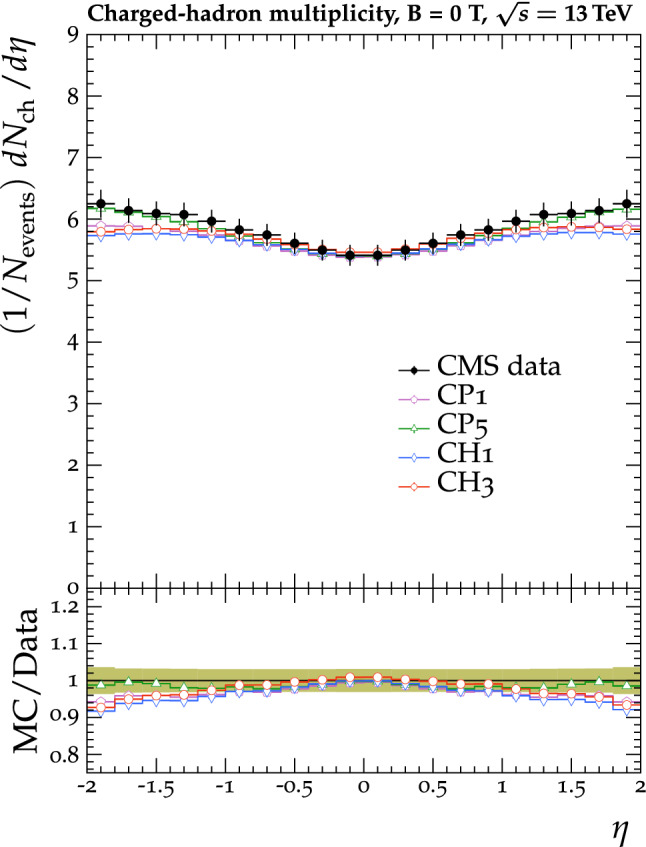
The normalized $\mathrm {d} N_{\text {ch}}/\mathrm {d} \eta$ of charged hadrons as a function of $\eta$ [27]. CMS MB data are compared with the predictions from ${\textsc {herwig}} \,7$, with the CH1 and CH3 tunes, and from ${\textsc {pythia}} \,8$, with the CP1 and CP5 tunes. The coloured band in the ratio plot represents the total experimental uncertainty in the data. The vertical bars on the points for the different predictions represent the statistical uncertainties

**Table 3:** Parameters of the central, “up”, and “down” variations of the CH3 tune

	CH3		
	Down	Central	Up
$p_{\bot ,\mathrm {0}}^\mathrm {min}$ ($\text {Ge}\text {V}$)	2.349	3.040	3.382
$b$	0.298	0.136	0.328
$\mu ^{2}$ ($\text {Ge}\text {V} ^{-2}$)	1.160	1.284	1.539
$p_{\text {reco}}$	0.641	0.471	0.191

**Fig. 9:**
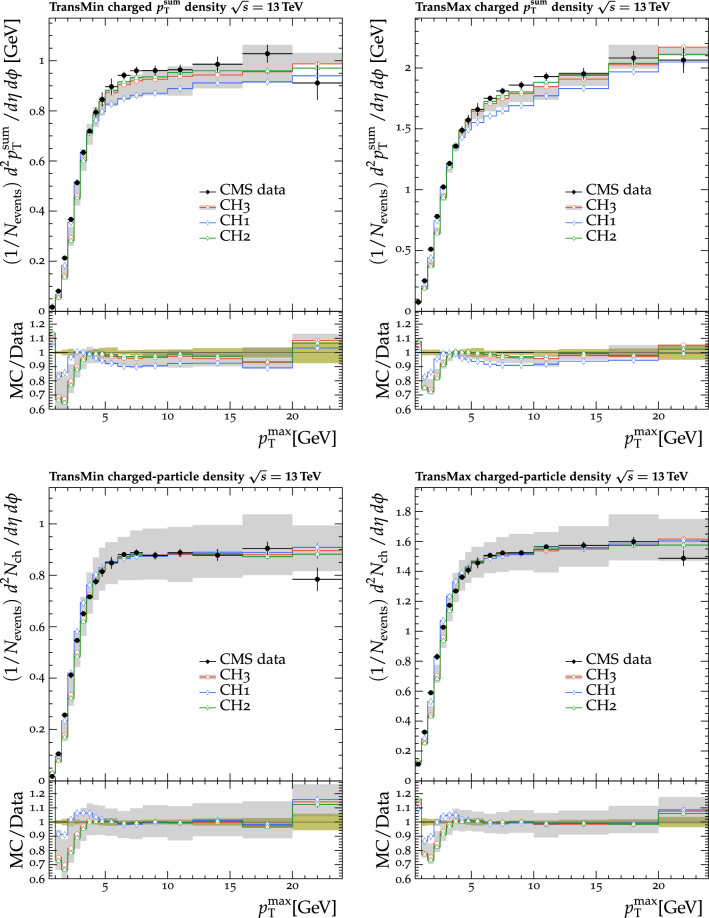
The $p_{\mathrm {T}} ^{\text {sum}}$ (upper) and $N_{\text {ch}}$ (lower) density distributions in the transMin (left) and transMax (right) regions, as a function of the $p_{\mathrm {T}}$ of the leading track, $p_{\mathrm {T}} ^{\text {max}}$ [24]. CMS MB data are compared with the predictions from ${\textsc {herwig}} \,7$, with the CH tunes. The coloured band in the ratio plot represents the total experimental uncertainty in the data. The vertical bars on the points for the different predictions represent the statistical uncertainties. The grey-shaded band corresponds to the envelope of the “up” and “down” variations of the CH3 tune

Figure 4 shows the normalized $N_{\text {ch}}$ and $p_{\mathrm {T}} ^{\text {sum}}$ densities as a function of $p_{\mathrm {T}} ^{\text {max}}$ using UE data at 7 TeV and compared with various tunes. In the transMax region, the predictions from the CH tunes describe the data well, with at most a 15% discrepancy at low $p_{\mathrm {T}} ^{\text {max}}$. In the transMin region, the predictions from all tunes deviate from the data at intermediate values of $p_{\mathrm {T}} ^{\text {max}} \approx 3\text {--}8\,\text {Ge}\text {V}$. The deviation is up to $\approx$10% for the CH2 and CH3 tunes, whereas the difference between data and the tunes $\text {SoftTune}$ and CH1 is larger than this. The prediction of CH1 deviates further from the data at lower values of $p_{\mathrm {T}} ^{\text {max}}$.

The predictions are compared with UE data at $\sqrt{s} =0.9\,\text {Te}\text {V}$ to normalized $p_{\mathrm {T}} ^{\text {sum}}$ densities in the transverse regions in Fig. 5. All tunes provide a similar prediction of the observables above $p_{\mathrm {T}} ^{\text {jet}} >4\,\text {Ge}\text {V}$, and agree with the data. Some differences are apparent between the predictions at low $p_{\mathrm {T}} ^{\text {jet}}$, with the tunes CH2 and CH3 providing a better description of the data compared to the tunes CH1 and $\text {SoftTune}$.

Figure 6 shows comparisons of the normalized $p_{\mathrm {T}} ^{\text {sum}}$ and $N_{\text {ch}}$ densities using tune predictions with UE data collected by the CDF experiment at the Fermilab Tevatron at $\sqrt{s} =1.96\,\text {Te}\text {V}$ [31]. The CH tunes describe the distributions in both transMin and transMax well, however the CH3 tune underestimates the $p_{\mathrm {T}} ^{\text {sum}}$ data somewhat at $p_{\mathrm {T}} ^{\text {max}} <10\,\text {Ge}\text {V}$, in both the transMin and transMax regions. Although these data were not used in deriving any of the tunes considered here, they validate that the energy dependence of the new tunes is correctly modelled. The tune $\text {SoftTune}$ overestimates the data by $\approx$5–15% in all distributions. Additional comparisons of the predictions of ${\textsc {herwig}} \,7$ with the various tunes using MB data from the ATLAS experiment, which were used in deriving $\text {SoftTune}$, are shown in Appendix A. One notable difference between the distribution of $\mathrm {d} N_{\text {ch}}/\mathrm {d} \eta$ shown in Fig. 2 and the one shown in Fig. 24 is that the former includes all charged particles, whereas the latter includes only charged particles with $p_{\mathrm {T}} > 500\,\text {Me}\text {V}$.

Based on the comparisons shown in this section, the tunes CH2 and CH3 both provide a similar description of the data, indicating that the choice between the two LO PDFs used for the simulation of MPI and remnant handling has little effect on the predictions. These two PDFs are both LO PDFs, but a value of $\alpha _\mathrm {S} (m_{\mathrm {\mathrm{Z}\,}}) =0.118$ is used in deriving the PDF used with CH2, and a value of $\alpha _\mathrm {S} (m_{\mathrm {\mathrm{Z}\,}}) =0.130$ is assumed for the PDF used with CH3. As stated in Sect. 3, $\alpha _\mathrm {S} (m_{\mathrm {\mathrm{Z}\,}}) =0.118$ is used in all parts of the ${\textsc {herwig}} \,7$ simulation for the three CH tunes. From Table 2, the $\chi ^{2}$/$N_{\text {dof}}$ for the tune CH2 is slightly lower than that for the tune CH3. However, the use of the LO PDF in the tune CH3, which was derived with $\alpha _\mathrm {S} (m_{\mathrm {\mathrm{Z}\,}}) =0.130$, is consistent with the value of $\alpha _\mathrm {S} (m_{\mathrm {\mathrm{Z}\,}})$ typically associated with LO PDFs and therefore is a preferred choice over the tune CH2. Both of the tunes CH2 and CH3 provide a better description of the data than the tune CH1, where the NNLO NNPDF3.1 PDF was used for the simulation of MPI and remnant handling. This suggests that the use of the LO NNPDF3.1 PDF is preferred in this aspect of the ${\textsc {herwig}} \,7$ simulation, even though the gluon PDF in both the LO and NNLO PDF sets are positive at low energy scales, as discussed earlier.

**Fig. 10:**
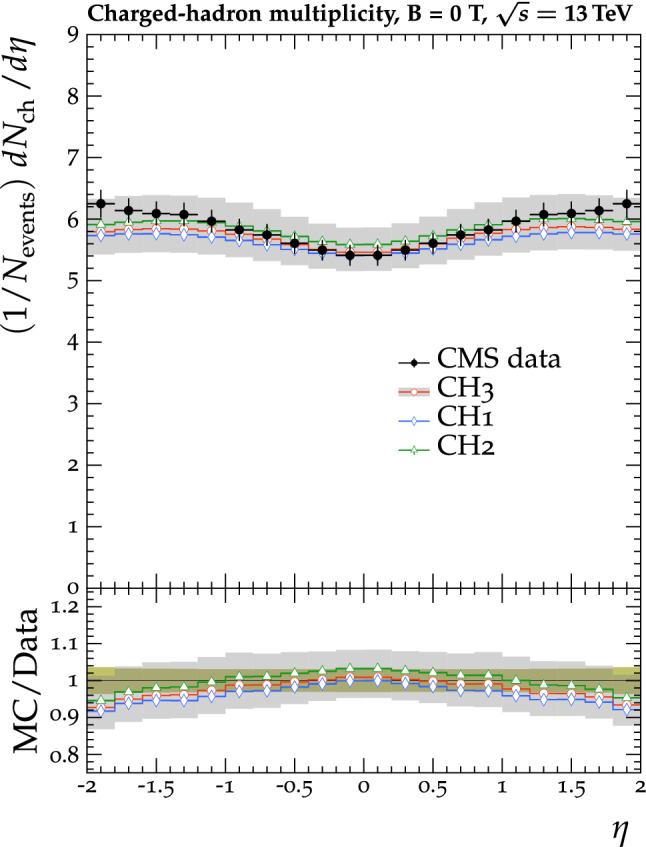
The normalized $\mathrm {d} N_{\text {ch}}/\mathrm {d} \eta$ of charged hadrons as a function of $\eta$ [27]. CMS MB data are compared with the predictions from ${\textsc {herwig}} \,7$, with the CH tunes. The coloured band in the ratio plot represents the total experimental uncertainty in the data. The vertical bars on the points for the different predictions represent the statistical uncertainties. The grey-shaded band corresponds to the envelope of the “up” and “down” variations of the CH3 tune

In Fig. 7 the normalized $N_{\text {ch}}$ and $p_{\mathrm {T}} ^{\text {sum}}$ density predictions of the UE data at $\sqrt{s} =13\,\text {Te}\text {V}$ show a comparison of the CH1 and CH3 tunes with those obtained from the ${\textsc {pythia}} \,8$ (version 8.230) using the tunes CP1 and CP5 [19]. The tune CH2 is not displayed, because its prediction is similar to the one of the tune CH3. The CP1 tune uses an LO NNPDF3.1 PDF set in all aspects of the ${\textsc {pythia}} \,8$ simulation, an $\alpha _\mathrm {S} (m_{\mathrm {\mathrm{Z}\,}})$ value of 0.130 in the simulation of MPI and hard scattering, and an $\alpha _\mathrm {S} (m_{\mathrm {\mathrm{Z}\,}})$ value of 0.1365 for the simulation of initial- and final-state radiation. The CP5 tune uses an NNLO PDF set with an $\alpha _\mathrm {S} (m_{\mathrm {\mathrm{Z}\,}})$ value of 0.118 in all aspects of simulation. The choice of the PDF set and $\alpha _\mathrm {S} (m_{\mathrm {\mathrm{Z}\,}})$ value in the CP5 tune is the same as the CH1  ${\textsc {herwig}} \,7$ tune. Although all the predictions show a reasonable agreement with the data in the plateau region of the UE distributions, the use of an LO PDF for MPI and remnant handling in CH3 provides a slightly improved description of the $p_{\mathrm {T}} ^{\text {sum}}$ data compared to using an NNLO PDF in CH1. This differs from the predictions of ${\textsc {pythia}} \,8$, where the use of an LO and NNLO PDF for simulating MPI give a similar description of the data in this region. Each prediction exhibits different behaviour at low $p_{\mathrm {T}} ^{\text {max}}$. None of the ${\textsc {herwig}} \,7$ or ${\textsc {pythia}} \,8$ tunes provides a perfect description of the data at low $p_{\mathrm {T}} ^{\text {max}}$, since they exhibit at least a 10% difference between any one of the tunes and the data. Figure 8 shows a similar comparison for the $\eta$ distribution of charged hadrons at 13$\,\text {Te}\text {V}$. The prediction from CP5 provides a better description of the data compared with the other tunes at larger values of $|\eta |$. The predictions from the ${\textsc {herwig}} \,7$ tunes show a closer behaviour to the CP1 tune in this distribution.

**Fig. 11:**
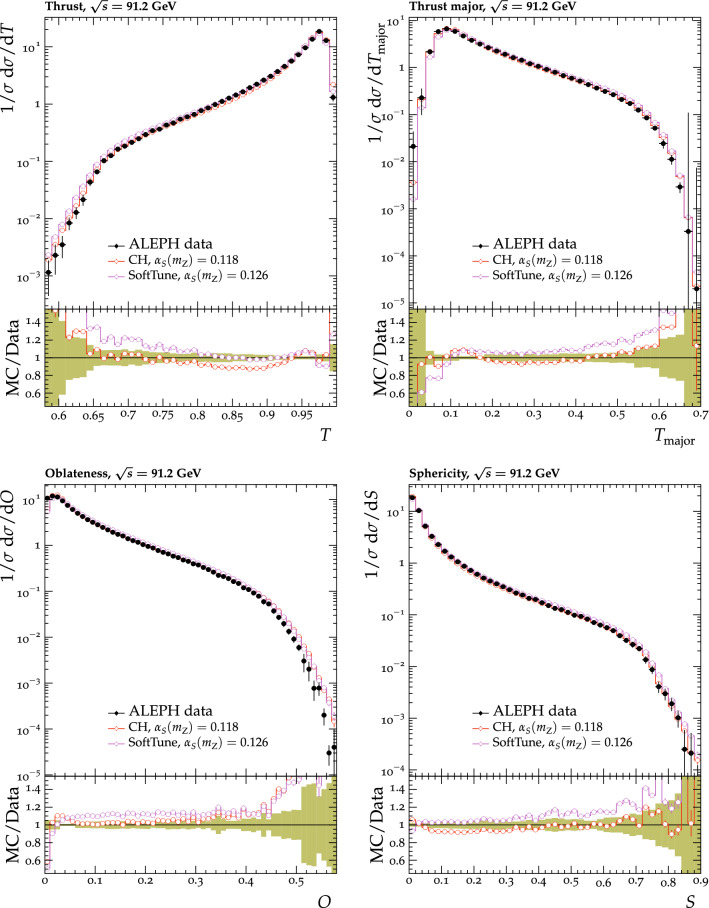
Normalized differential cross sections for $\mathrm{e}^-$ $\mathrm{e}^+$ [32] as a function of the variables $T$ (upper left), $T_{\mathrm {major}}$ (upper right), $O$ (lower left), and $S$ (lower right) for ALEPH data at $\sqrt{s} =91.2\,\text {Ge}\text {V}$. ALEPH data are compared with the predictions from ${\textsc {herwig}} \,7$ using the $\text {SoftTune}$ and CH tunes. The coloured band in the ratios of the different predictions from simulation to the data represents the total experimental uncertainty in the data

**Fig. 12:**
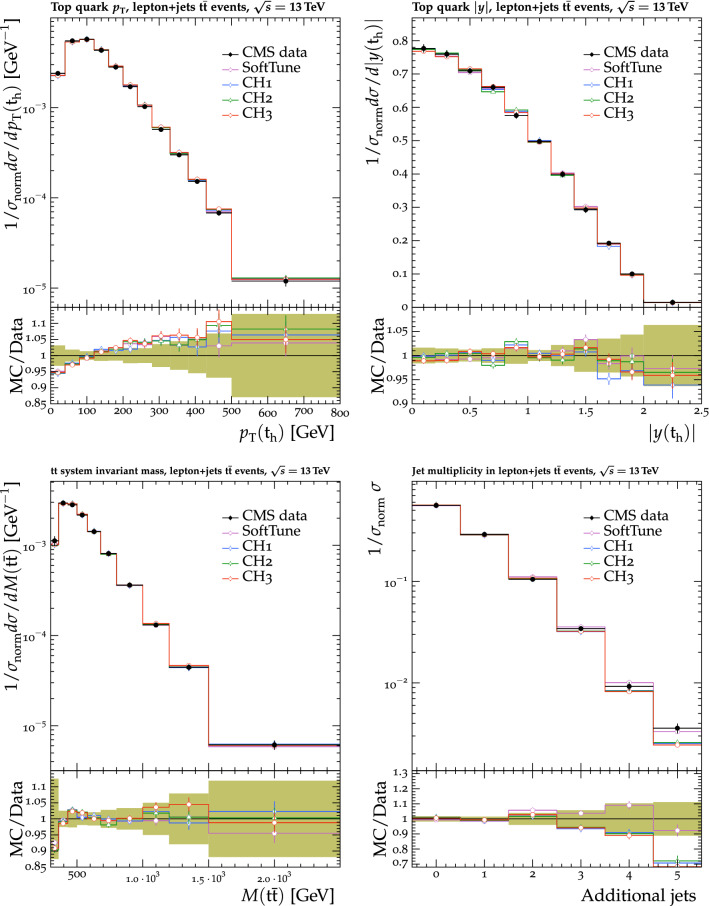
The differential cross sections are shown as functions of: the $p_{\mathrm {T}}$ (upper left) and rapidity (upper right) of the hadronically decaying top quark; the invariant mass of the $\mathrm{t}{\bar{\mathrm{t}}}$ system (lower left); the additional jet multiplicity (lower right) [38]. CMS $\mathrm{t}{\bar{\mathrm{t}}}$ data are compared with the predictions from powheg  + ${\textsc {herwig}} \,7$, with the $\text {SoftTune}$, CH1, CH2, and CH3 tunes. The coloured band in the ratio plot represents the total experimental uncertainty in the data. The vertical bars on the points for the different predictions represent the statistical uncertainties

**Fig. 13:**
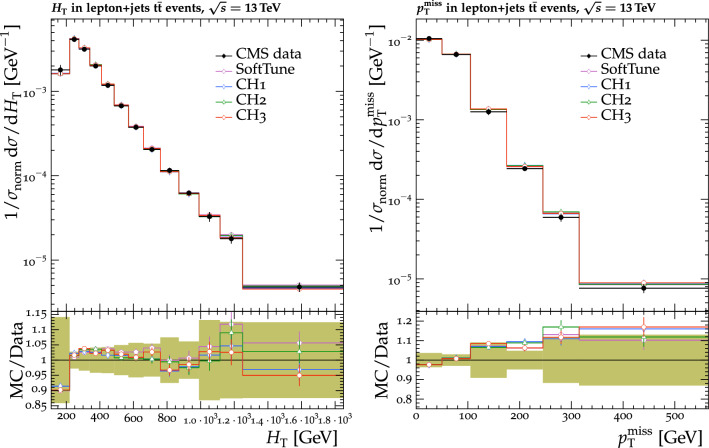
The differential cross sections are shown as functions of $H_{\mathrm {T}}$ (left) and $p_{\mathrm {T}} ^\text {miss}$ (right) [41]. CMS $\mathrm{t}{\bar{\mathrm{t}}}$ data are compared with the predictions from powheg  + ${\textsc {herwig}} \,7$, with the $\text {SoftTune}$, CH1, CH2, and CH3 tunes. The coloured band in the ratio plot represents the total experimental uncertainty in the data. The vertical bars on the points for the different predictions represent the statistical uncertainties

**Fig. 14:**
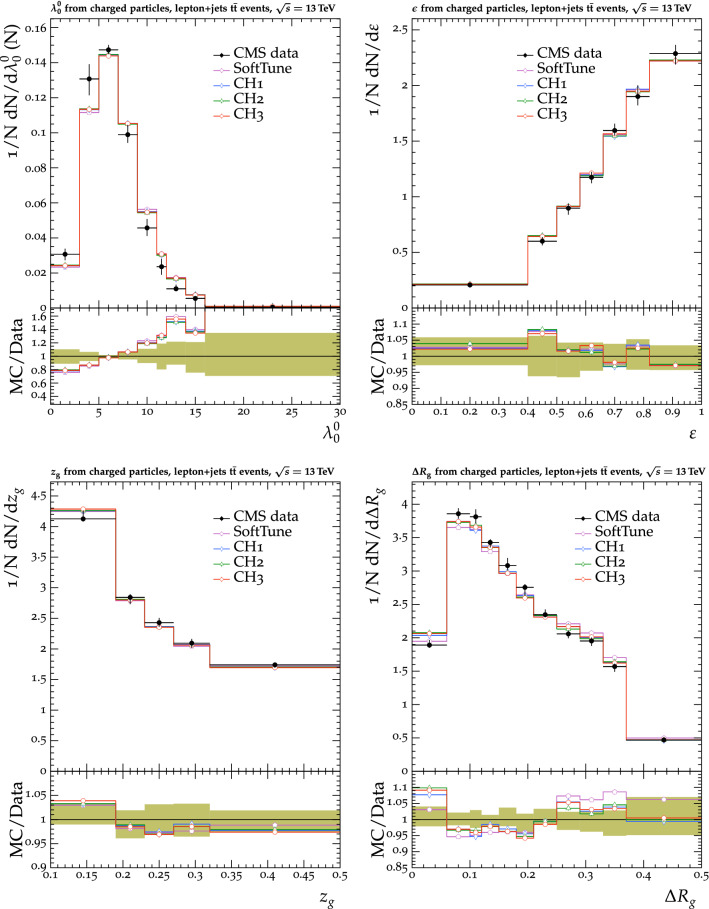
The normalized jet substructure observables in single-lepton events: the charged-particle multiplicity (upper left); the eccentricity (upper right); the groomed momentum fraction (lower left); and the angle between the groomed subjects (lower right) [42]. CMS $\mathrm{t}{\bar{\mathrm{t}}}$ data are compared with the predictions from powheg  + ${\textsc {herwig}} \,7$, with the $\text {SoftTune}$, CH1, CH2, and CH3 tunes. The coloured band in the ratio plot represents the total experimental uncertainty in the data. The vertical bars on the points for the different predictions represent the statistical uncertainties

**Fig. 15:**
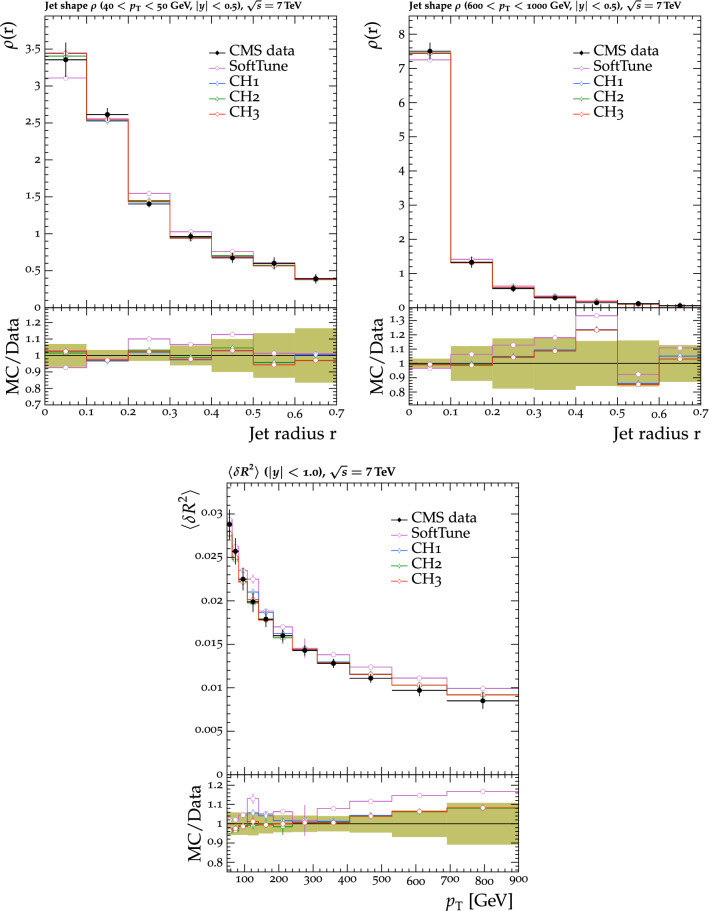
The differential jet shape $\rho (\mathrm {r})$ (upper left and right) and the second moment of the jet transverse width $\langle \delta R^2\rangle$ in inclusive jet events [43]. CMS inclusive jet data are compared with the predictions from ${\textsc {herwig}} \,7$, with the $\text {SoftTune}$ and CH tunes. The coloured band in the ratio plot represents the total experimental uncertainty in the data. The vertical bars on the points for the different predictions represent the statistical uncertainties

**Fig. 16:**
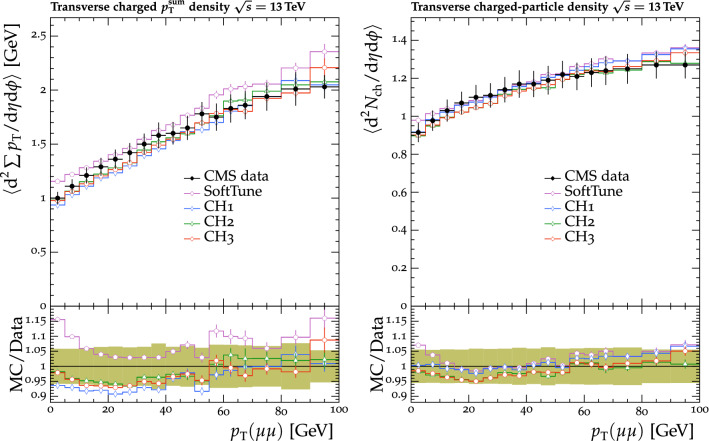
The $p_{\mathrm {T}} ^{\text {sum}}$ (left) and $N_{\text {ch}}$ (right) density distributions in the transverse region, as a function of the $p_{\mathrm {T}}$ of the two muons, $p_{\mathrm {T}} (\mu \mu )$ [45]. The transverse region is defined with respect to $p_{\mathrm {T}} (\mu \mu )$, where the two muons are required to have an invariant mass close the the mass of the Z boson. CMS Z boson data are compared with the predictions from MadGraph 5_amc@nlo + ${\textsc {herwig}} \,7$, with the $\text {SoftTune}$ and CH tunes. The coloured band in the ratio plot represents the total experimental uncertainty in the data. The vertical bars on the points for the different predictions represent the statistical uncertainties

**Fig. 17:**
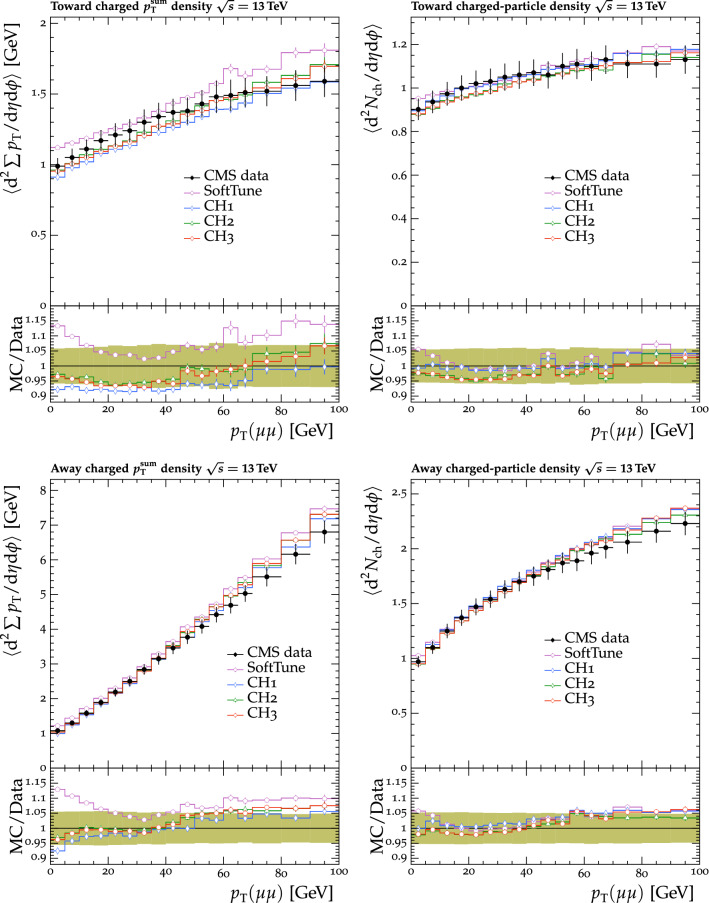
The $p_{\mathrm {T}} ^{\text {sum}}$ (left) and $N_{\text {ch}}$ (right) density distributions in the toward (upper), and away (lower) regions, as a function of the $p_{\mathrm {T}}$ of the two muons, $p_{\mathrm {T}} (\mu \mu )$ [45]. The toward and away regions are defined with respect to $p_{\mathrm {T}} (\mu \mu )$, where the two muons are required to have an invariant mass close the the mass of the Z boson. CMS Z boson data are compared with the predictions from MadGraph 5_amc@nlo + ${\textsc {herwig}} \,7$, with the $\text {SoftTune}$ and CH tunes. The coloured band in the ratio plot represents the total experimental uncertainty in the data. The vertical bars on the points for the different predictions represent the statistical uncertainties

**Fig. 18:**
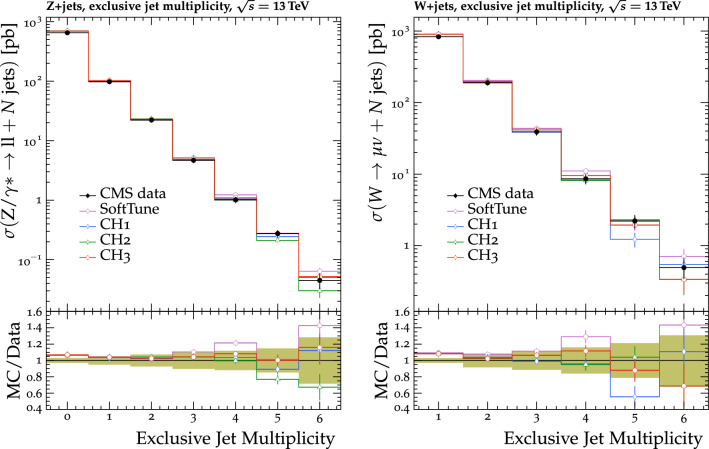
The exclusive jet multiplicity in Z (left) and W (right) boson events, measured by CMS at $\sqrt{s} =13\,\text {Te}\text {V}$ [46, 47]. CMS Z boson and W boson data are compared with the predictions from MadGraph 5_amc@nlo + ${\textsc {herwig}} \,7$, with the $\text {SoftTune}$ and CH tunes. The coloured band in the ratio plot represents the total experimental uncertainty in the data. The vertical bars on the points for the different predictions represent the statistical uncertainties

**Fig. 19:**
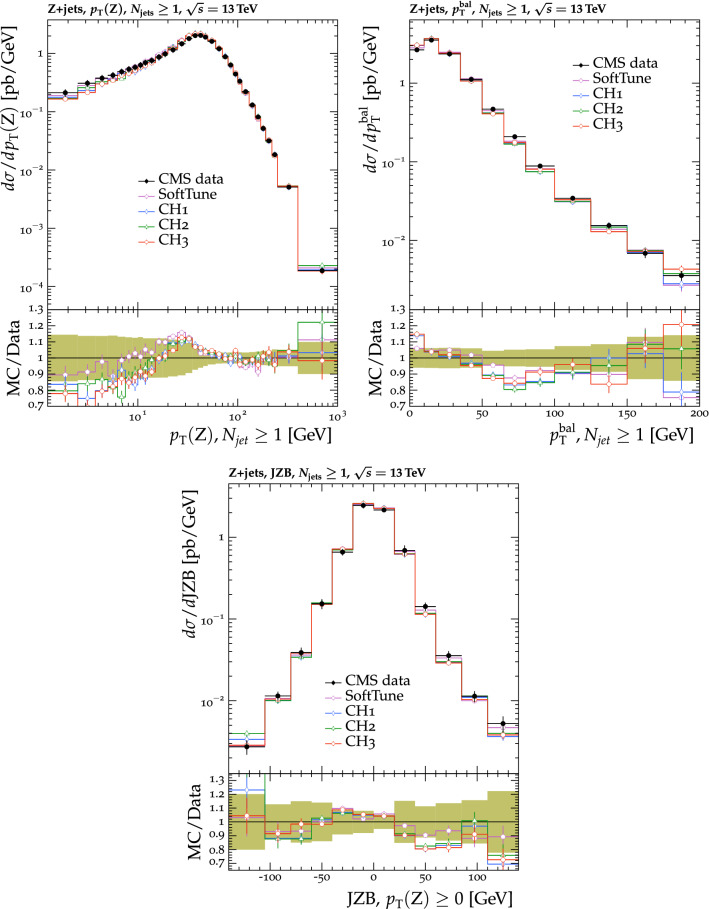
Differential cross sections as a function of $p_{\mathrm {T}} (\mathrm{Z}\,)$ (upper left), $p_{\mathrm {T}} ^{\text {bal}}$ (upper right), and $\mathrm {JZB}$ (lower) [46]. CMS Z boson data are compared with the predictions from MadGraph 5_amc@nlo + ${\textsc {herwig}} \,7$, with the $\text {SoftTune}$ and CH tunes. The coloured band in the ratio plot represents the total experimental uncertainty in the data. The vertical bars on the points for the different predictions represent the statistical uncertainties

## Uncertainties in the HERWIG 7 tunes

Alternative tunes are derived in this section that provide an approximation to the uncertainties in the parameters of the tune CH3. These are obtained from the eigentunes provided by professor. These eigentunes are variations of the tuned parameters along the maximally independent directions in the parameter space by an amount corresponding to a change in the $\chi ^{2}$ ($\varDelta \chi ^{2}$) equal to the optimal $\chi ^{2}$ of the fit. Because a change $\varDelta \chi ^{2}$ in Eq. (5) does not result in a variation with a meaningful statistical interpretation, the value of $\varDelta \chi ^{2}$ is chosen in an empirical way. The change $\varDelta \chi ^{2} =\chi ^{2}$, which is suggested by the professor Collaboration, results in variations that are similar in magnitude to the uncertainties in the fitted data points and judged to provide a reasonable set of variations that reflect the combined statistical and systematic uncertainty in the model parameters. A consequence of this adopted procedure is that the uncertainty may not necessarily cover the data in every bin. If the uncertainties in the fitted data points were uncorrelated between themselves, then the magnitude of the uncertainties in the data points depends on their bin widths. For the data used in the fit, the uncertainties are typically dominated by uncertainties that are correlated between the bins. However, the uncertainties in the data points at high $p_{\mathrm {T}} ^{\text {max}}$ and $p_{\mathrm {T}} ^{\text {jet}}$, e.g. $p_{\mathrm {T}} ^{\text {max}} \gtrsim 10\,\text {Ge}\text {V}$ for the UE observables at $\sqrt{s} =13\,\text {Te}\text {V}$, are dominated by statistical uncertainties, which are uncorrelated between bins. This introduces some dependence of the eigentunes on the bin widths of the data used in the fit.

The variations of the tunes provided by the eight eigentunes are reduced to two variations, as explained below, one “up” and one “down” variation. The “up” variation is obtained by considering the positive differences in each bin between each eigentune and the central prediction of the CH3 tune for the distributions used in the tuning procedure. The difference for each eigentune is summed in quadrature. Similarly, the “down” variation is obtained by considering the negative differences between the eigentunes and the central predictions. The two variations are then fitted, using the same procedure described in Sect. 3 to obtain a set of tune parameters that describe these two variations. The parameters of the two variations are shown in Table 3. The values of each parameter of the variations do not necessarily encompass the corresponding values of the CH3 tune, as a result of the method of determining the variations from the differences between several eigentunes. The two variations accurately replicate the combination of all eigentunes, i.e. the sum in quadrature of all positive or negative differences with respect to the central prediction. By using these variations, the uncertainties in the tune CH3 are estimated by considering only two variations of the tune parameters, rather than eight variations. However, the correlations between bins of an observable for each of the eight individual variations are not known when considering only the “up” and “down” variations.

Figures 9 (normalized $p_{\mathrm {T}} ^{\text {sum}}$ and $N_{\text {ch}}$ densities) and 10 (normalized $\mathrm {d} N_{\text {ch}}/\mathrm {d} \eta$) show predictions from the CH tunes. The grey-shaded band corresponds to the envelope of the “up” and “down” variations, for the UE and MB observables used in the tuning procedure. The differences between the CH1 and CH2 predictions and those from CH3 are within the uncertainty of CH3, except for a small deviation at low $p_{\mathrm {T}} ^{\text {max}}$.

## Comparison with LEP data

${\textsc {herwig}} \,7$ predictions are obtained in this section for event shape observables measured in LEP electron-positron collisions at $\sqrt{s} =91.2\,\text {Ge}\text {V}$. The predictions are obtained using NLO MEs implemented within ${\textsc {herwig}} \,7$. Figure 11 shows the thrust ($T$), thrust major ($T_{\mathrm {major}}$), oblateness ($O$), and sphericity ($S$) observables as measured by the ALEPH Collaboration [32].

Because these observables are measured in collisions with a lepton-lepton initial state, the difference in choice of PDF and parameters of the MPI model in the three CH tunes has no effect on the predictions. Similarly, the only difference between the CH tunes and $\text {SoftTune}$ is in the value of $\alpha _\mathrm {S} (m_{\mathrm {\mathrm{Z}\,}})$. The value of $\alpha _\mathrm {S} (m_{\mathrm {\mathrm{Z}\,}}) =0.118$ is used in the CH tunes, and is consistent with the value used by the PDF set for the hard process and the PS when simulating proton-proton collisions. A set of next-to-leading corrections to soft gluon emissions can be incorporated in the PS by using two-loop running of $\alpha _\mathrm {S}$ and including the Catani–Marchesini–Webber rescaling [33] of $\alpha _\mathrm {S} (m_{\mathrm {\mathrm{Z}\,}})$ from $\alpha _\mathrm {S} (m_{\mathrm {\mathrm{Z}\,}}) =0.118$ to $\alpha _\mathrm {S} (m_{\mathrm {\mathrm{Z}\,}}) =0.1262$, which corresponds to the value of $\alpha _\mathrm {S} (m_{\mathrm {\mathrm{Z}\,}})$ used in $\text {SoftTune}$ [34].

The CH tunes underestimate the number of events with $0.80< T < 0.95$, whereas $\text {SoftTune}$ predicts too many isotropic events with lower values of $T <0.8$ and with higher values of $S >0.4$. The CH tune provides a better overall description of the $T_{\mathrm {major}}$ observable compared with $\text {SoftTune}$. Both tunes predict too many planar events, as can be seen at larger values of $O$; however, the CH tune provides a better description of the data at smaller values of $O$.

## Comparison with top quark pair production data

Predictions using the ${\textsc {herwig}} \,7$ tunes are compared in this section with observables measured in data containing top quark pairs.

The powheg v2 generator is used to perform ME calculations in the hvq mode [35] at NLO accuracy in QCD. In the powheg ME calculations, a value of $\alpha _\mathrm {S} (m_{\mathrm {\mathrm{Z}\,}}) =0.118$ with a two-loop evolution of $\alpha _\mathrm {S}$ is used, along with the NNPDF 3.1 NNLO PDF set, derived with a value of $\alpha _\mathrm {S} (m_{\mathrm {\mathrm{Z}\,}}) =0.118$. The ME calculations are interfaced with ${\textsc {herwig}} \,7$ for the simulation of the UE and PS. The mass of the top quark is set to $m_{\mathrm{t}\,} = 172.5\,\text {Ge}\text {V}$, and the value of the $h_{\text {damp}}$ parameter, which controls the matching between the ME and PS, is set to $1.379~m_{\mathrm{t}\,}$. The value of $h_{\text {damp}}$ in powheg was derived from a fit to $\mathrm{t}{\bar{\mathrm{t}}}$ data in the dilepton channel at $\sqrt{s} =8\,\text {Te}\text {V}$, where powheg was interfaced with ${\textsc {pythia}} \,8$ using the CP5 tune [19, 36].

Samples are generated with the different ${\textsc {herwig}} \,7$ tunes that use the same parton-level events for each tune. For generating NLO matched samples such as these, an NLO (or NNLO) PDF set may be desirable for the simulation of the hard process. In Ref. [37], it is then advocated that the same PDF set and $\alpha _\mathrm {S} (m_{\mathrm {\mathrm{Z}\,}})$ value should be used in the PS. However, one can still choose an LO PDF set for the simulation of the MPI and remnant handling in this case, such as the choices in the tunes CH2 and CH3. This configuration of PDF sets is not possible in pythia.

First, kinematic properties of the $\mathrm{t}{\bar{\mathrm{t}}}$ system are compared with $\sqrt{s} =13\,\text {Te}\text {V}$ CMS data in the single-lepton channel [38]. Figure 12 presents normalized differential cross sections as functions of the $p_{\mathrm {T}}$ and rapidity *y* of the particle-level hadronically decaying top quark. The invariant mass of the reconstructed $\mathrm{t}{\bar{\mathrm{t}}}$ system and the number of additional jets with $p_{\mathrm {T}} > 30\,\text {Ge}\text {V}$ in the event are also shown, where the jets are reconstructed using the anti-$k_{\mathrm {T}}$ algorithm [39, 40] with a distance parameter of 0.4. Normalized cross sections as a function of global event variables, namely $H_{\mathrm {T}}$, the scalar $p_{\mathrm {T}}$ sum of all jets, and $p_{\mathrm {T}} ^\text {miss}$, the magnitude of the missing transverse momentum vector [41] are shown in Fig. 13.

The predictions from the different simulations are mostly compatible with each other, indicating a small effect of the tune on these observables. The only notable difference is seen in the additional jet multiplicity, originating from the smaller $\alpha _\mathrm {S} (m_{\mathrm {\mathrm{Z}\,}})$ value used in the simulations with ${\textsc {herwig}} \,7$  CH tunes. The simulated events with the CH tunes describe the CMS data well up to 4 additional jets, but slightly underestimate the multiplicity for a higher number of jets. The differences between the predictions with the CH tunes and the tune $\text {SoftTune}$ are comparable with the typical size of the theoretical uncertainties in the ME calculation, as studied in Ref. [36].

Next, jet substructure observables are compared to $\sqrt{s} =13\,\text {Te}\text {V}$ CMS data in the single-lepton channel [42]. Normalized number of jets as a function of four variables with relatively low correlations amongst themselves are shown in Fig. 14. The variables presented are the charged-particle multiplicity ($\lambda _0^0$), the eccentricity ($\varepsilon$) calculated from the charged jet constituents, the groomed momentum fraction ($z_\mathrm {g}$), and the angle between the groomed subjets ($\varDelta R_\mathrm {g}$).

The choice of tune has little effect on most of the jet substructure observables. All choices of ${\textsc {herwig}} \,7$ tune overestimate $\lambda _0^0$, which was also observed in Ref. [42]. The predictions for $\varepsilon$ and $z_\mathrm {g}$ distributions agree closely with the data in all cases. The $\varDelta R_\mathrm {g}$ spectrum at very low values is somewhat less well described by the simulation employing the CH tunes, whereas for high values the description is better for the CH tune samples than with $\text {SoftTune}$. Since the $\varDelta R_\mathrm {g}$ observable is strongly dependent on the amount of final-state radiation [42], the difference comes mostly from the choice of $\alpha _\mathrm {S} (m_{\mathrm {\mathrm{Z}\,}})$, with the choice of $\alpha _\mathrm {S} (m_{\mathrm {\mathrm{Z}\,}})$ in the CH tunes preferred to that in $\text {SoftTune}$.

## Comparisons with inclusive jet events

The predictions of ${\textsc {herwig}} \,7$ with the various tunes for inclusive jet production are investigated in this section. In particular, the substructure of the jets is considered. Events are generated with the LO QCD two-to-two MEs implemented in ${\textsc {herwig}} \,7$. Although a comparison of the substructure of jets in $\mathrm{t}{\bar{\mathrm{t}}}$ events was already presented in Sect. 7, the comparison based on inclusive jet events is complementary because it probes a wider range of jet $p_{\mathrm {T}}$.

Figure 15 shows the differential jet shape, $\rho (\mathrm {r})$, as measured by the CMS experiment at $\sqrt{s} =7\,\text {Te}\text {V}$ [43] for two bins of ranges of jet $p_{\mathrm {T}}$ ($p_{\mathrm {T}} ^{\text {jet}}$): $40< p_{\mathrm {T}} ^{\text {jet}} < 50 \,\text {Ge}\text {V}$ and $600< p_{\mathrm {T}} ^{\text {jet}} < 1000 \,\text {Ge}\text {V}$. The observable $\rho (\mathrm {r})$ is defined as the average fraction of the $p_{\mathrm {T}}$ of the jet constituents contained inside an annulus with inner radius $\mathrm {r}-0.1$ and outer radius $\mathrm {r}+0.1$. The second moment of the jet transverse width, $\langle \delta R^2\rangle$, is also shown. The jets are clustered with the anti-$k_{\mathrm {T}}$ algorithm with a distance parameter of 0.7 for the jet shape observables, and 0.5 for the $\langle \delta R^2\rangle$ observable. The predictions from the three CH tunes are very similar for all distributions, and agree with the data. On the other hand, the prediction from $\text {SoftTune}$ differs from the CH tunes, and also does not agree well with the $\langle \delta R^2\rangle$ distribution in data.

Additional comparisons of the predictions for various tunes of ${\textsc {herwig}} \,7$ tunes with the substructure of jets collected by the ATLAS experiment are shown in Appendix B.

## Comparison with Z  and W  boson production data

In this section, the performance of the ${\textsc {herwig}} \,7$ tunes is compared with $\sqrt{s} =13\,\text {Te}\text {V}$ data on Z and W boson production. Predictions for Z and W boson production are obtained with MadGraph 5_amc@nlo v2.6.7 [9] for ME calculations at NLO, which are interfaced with ${\textsc {herwig}} \,7$ using the the FxFx merging scheme [44], with the merging scale set to 30$\,\text {Ge}\text {V}$. Up to two additional partons in the final state are included in the NLO ME calculations. The PDF in the ME calculations is NNPDF 3.1 NNLO, and the value of $\alpha _\mathrm {S} (m_{\mathrm {\mathrm{Z}\,}})$ in the ME calculations is set to $\alpha _\mathrm {S} (m_{\mathrm {\mathrm{Z}\,}}) =0.118$ in all the predictions considered here.

First, the $p_{\mathrm {T}} ^{\text {sum}}$ and $N_{\text {ch}}$ distributions characterizing the UE in Z boson production [45] are compared to simulation in Figs. 16 and 17. Events are required to have two muons with an invariant mass between 81 and 101$\,\text {Ge}\text {V}$ to select events within the Z boson mass peak. The $p_{\mathrm {T}} ^{\text {sum}}$ and $N_{\text {ch}}$ distributions are measured in the transverse region as shown in Fig. 16, and in the toward and away regions as shown in Fig. 17, in analogy to the corresponding distributions measured in MB data introduced in Sect. 3. The regions are defined with respect to the $p_{\mathrm {T}}$ of the Z boson, calculated from the $p_{\mathrm {T}}$ of the two muons. The CH tunes describe the data well, and are typically similar to each other. However, the configuration with $\text {SoftTune}$ fails to give a simultaneous description of the $p_{\mathrm {T}} ^{\text {sum}}$ and $N_{\text {ch}}$ distributions in any region at low $p_{\mathrm {T}} (\mu \mu )$.

Next, the exclusive jet multiplicity distributions in Z and W boson events are shown in Fig. 18 [46, 47]. Events in the Z boson sample contain at least two electrons or muons with $p_{\mathrm {T}} >20\,\text {Ge}\text {V}$ and $|\eta |<2.4$, and the invariant mass of the two highest $p_{\mathrm {T}}$ electrons or muons must have an invariant mass within 20$\,\text {Ge}\text {V}$ of the Z boson mass. In the W boson measurement, only final states with a muon of $p_{\mathrm {T}} >25\,\text {Ge}\text {V}$ and $|\eta |<2.4$ are considered. The transverse mass of the W boson candidate, defined as $m_{\mathrm {T}} = \sqrt{\smash [b]{2p_{\mathrm {T}} ^\mu p_{\mathrm {T}} ^\text {miss} [1-\cos (\varDelta \phi _{\mu ,{\vec p}_{\mathrm {T}}^{\text {miss}}})]}}$, where $\cos (\varDelta \phi _{\mu ,{\vec p}_{\mathrm {T}}^{\text {miss}}})$ is the difference in azimuthal angle between the direction of the muon momentum and $p_{\mathrm {T}} ^\text {miss}$, must satisfy $m_{\mathrm {T}} >50\,\text {Ge}\text {V}$. In both Z and W events jets are reconstructed using the anti-$k_{\mathrm {T}}$ algorithm with a distance parameter of 0.4, and are required to satisfy $p_{\mathrm {T}} >30\,\text {Ge}\text {V}$ and $|y |<2.4$. Jets must also be separated from any lepton by $\sqrt{\smash [b]{(\varDelta \eta )^2+(\varDelta \phi )^2}} >0.4$, where $\phi$ is in radians. The jet multiplicity is well described by all tunes in both Z and W boson events at both low multiplicities, where the ME calculations dominate, and high multiplicities, where the PS is important.

Finally, in Fig. 19, the $p_{\mathrm {T}} (\mathrm{Z}\,)$ and $p_{\mathrm {T}} ^{\text {bal}}$ distributions are shown, both for final states containing at least one additional jet. The $p_{\mathrm {T}} ^{\text {bal}}$ variable is defined as $p_{\mathrm {T}} ^{\text {bal}} = |{\vec p}_{\mathrm {T}} (\mathrm{Z}\,) +\sum _{\mathrm {jets}}{\vec p}_{\mathrm {T}} (\mathrm {j}) |$. The so-called jet-Z balance ($\mathrm {JZB}$) variable, defined as $\mathrm {JZB} = |\sum _{\mathrm {jets}}{\vec p}_{\mathrm {T}} (\mathrm {j}) |-|{\vec p}_{\mathrm {T}} (\mathrm{Z}\,) |$, is also shown in Fig. 19. All distributions are measured for events with at least one additional jet. The $p_{\mathrm {T}} (\mathrm{Z}\,)$ predictions for all tunes are similar for $p_{\mathrm {T}} (\mathrm{Z}\,) >30\,\text {Ge}\text {V}$, where the predictions are driven by the ME calculations. At lower $p_{\mathrm {T}} (\mathrm{Z}\,)$, where events contain additional hadronic activity that is not clustered into jets, the predictions with the CH tunes are similar to each other, and differ slightly from the prediction with $\text {SoftTune}$, which provides a closer description of the data at very low $p_{\mathrm {T}} (\mathrm{Z}\,) <10\,\text {Ge}\text {V}$. The $p_{\mathrm {T}} ^{\text {bal}}$ and $\mathrm {JZB}$ distributions are also sensitive to additional hadronic activity not clustered into jets. For $p_{\mathrm {T}} ^{\text {bal}}$, all tunes are compatible with each other, except at $p_{\mathrm {T}} ^{\text {bal}} <10\,\text {Ge}\text {V}$, where the prediction with $\text {SoftTune}$ differs from the predictions with the CH tunes. The $\mathrm {JZB}$ distributions are well described by all the predictions.

## Summary

Three new tunes for the multiple-parton interaction (MPI) model of the ${\textsc {herwig}} \,7$ (version 7.1.4) generator have been derived from minimum-bias (MB) data collected by the CMS experiment. All of the CH (“CMS herwig ”) tunes, CH1, CH2, and CH3, are based on the next-to-next-to-leading-order (NNLO) NNPDF 3.1 PDF set for the simulation of the parton shower (PS) in ${\textsc {herwig}} \,7$; the value of the strong coupling at a scale equal to the Z boson mass is $\alpha _\mathrm {S} (m_{\mathrm {\mathrm{Z}\,}}) =0.118$ with a two-loop evolution of $\alpha _\mathrm {S}$. The configuration of the tunes differs in the PDF used for the simulation of MPI and beam remnants. The tune CH1 uses the same NNLO PDF set for these aspects of the ${\textsc {herwig}} \,7$ simulation, whereas CH2 and CH3 use leading-order (LO) versions of the PDF set. The tune CH2 is based on an LO PDF set that was derived assuming $\alpha _\mathrm {S} (m_{\mathrm {\mathrm{Z}\,}}) =0.118$, and CH3 on an LO PDF set assuming $\alpha _\mathrm {S} (m_{\mathrm {\mathrm{Z}\,}}) =0.130$.

The parameters of the MPI model were optimized for each tune with the professor framework to describe the underlying event (UE) in MB data collected by CMS. The predictions using the tune CH2 or CH3 provide a better description of the data than those using CH1 or $\text {SoftTune}$. Furthermore, the differences in the predictions of CH2 and CH3 are observed to be small. The configuration of PDF sets in the tune CH3, where the LO PDF used for the simulation of MPI, was derived with a value of $\alpha _\mathrm {S} (m_{\mathrm {\mathrm{Z}\,}})$ typically associated with LO PDF sets, is the preferred choice over CH2. Two alternative tunes representing the uncertainties in the fitted parameters of CH3 are also derived, based on the tuning procedure provided by professor.

Predictions using the three CH tunes are compared with a range of data beyond MB events: event shape data from LEP; proton-proton data enriched in top quark pairs, Z bosons and W bosons; and inclusive jet data. This validated the performance of ${\textsc {herwig}} \,7$ using these tunes against a wide range of data sets sensitive to various aspects of the modelling by ${\textsc {herwig}} \,7$, and in particular the modelling of the UE. The event shape observables measured at LEP, which are sensitive to the modelling of final-state radiation, are well described by ${\textsc {herwig}} \,7$ with the new tunes. Predictions using the new tunes are also shown to describe the UE in events containing Z bosons, demonstrating the universality of the UE modelling in ${\textsc {herwig}} \,7$. The kinematics of top quark events, and the modelling of jets in $\mathrm{t}{\bar{\mathrm{t}}}$ , Z boson, W boson, and inclusive jet data are also well described by predictions using the new tunes. In general, predictions with the new CH tunes derived in this paper provide a better description of measured observables than those using $\text {SoftTune}$, the default tune available in ${\textsc {herwig}} \,7$.

**Fig. 20:**
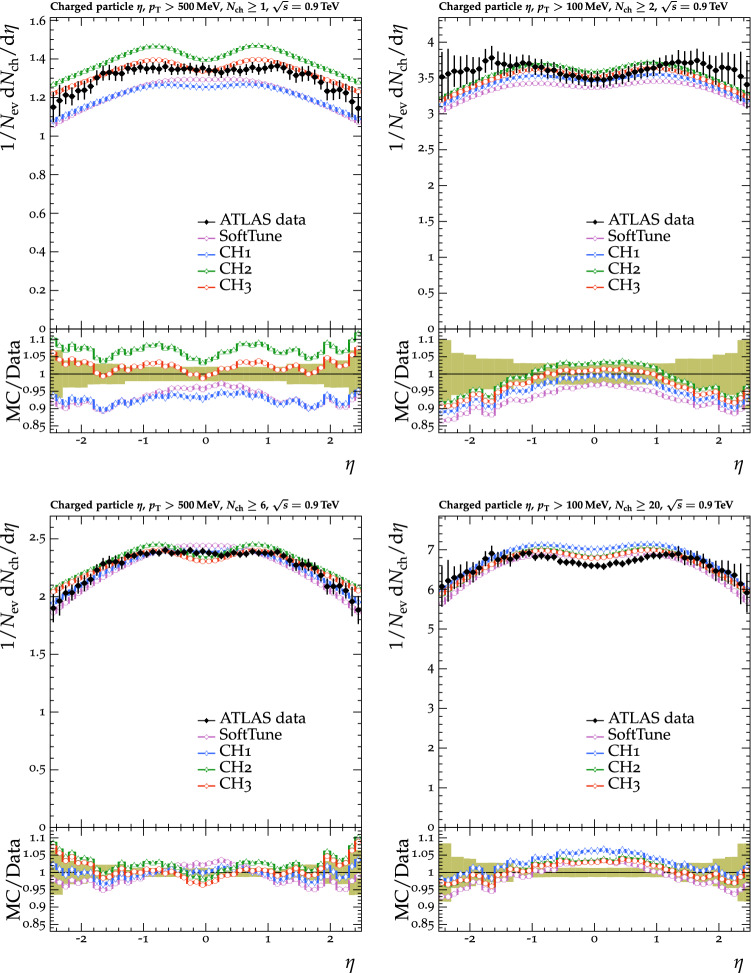
Normalized plots [17] for the pseudorapidity of charged particles for $N_{\text {ch}} \ge 1$ (upper left), and $N_{\text {ch}} \ge 6$ (lower left), for charged particles with $p_{\mathrm {T}} >500\,\text {Me}\text {V}$. The figure on the upper right shows a similar distribution for $N_{\text {ch}} \ge 2$, and the lower right for $N_{\text {ch}} \ge 20$, where the charged particles have $p_{\mathrm {T}} >100\,\text {Me}\text {V}$. ATLAS MB data are compared with the predictions from ${\textsc {herwig}} \,7$, with the $\text {SoftTune}$ and CH tunes. The coloured band in the ratio plot represents the total experimental uncertainty in the data. The vertical bars on the points for the different predictions represent the statistical uncertainties

## Figures and Tables

**Fig. 21:**
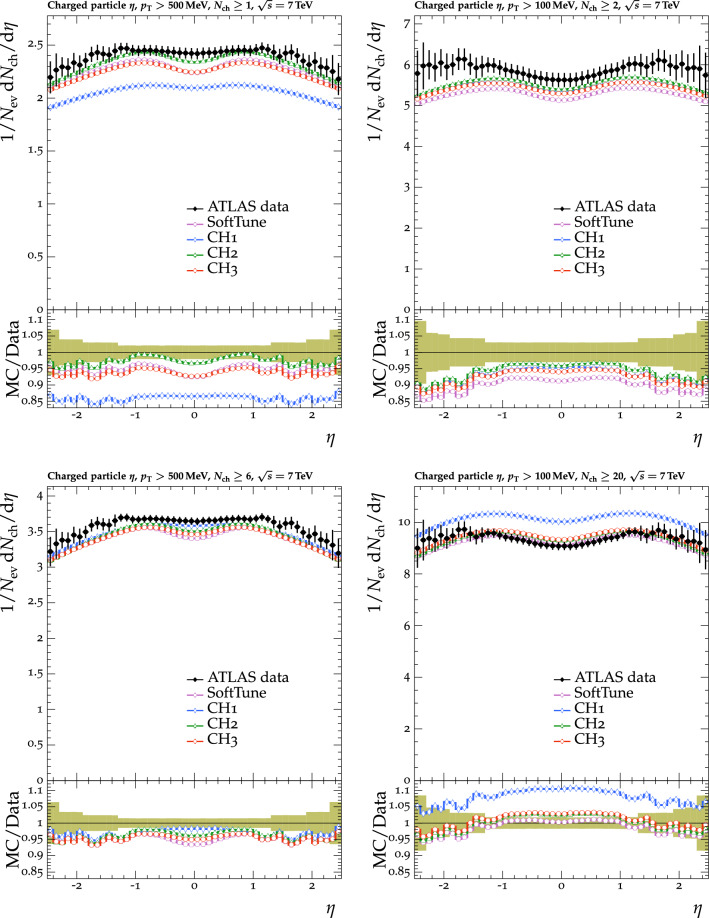
Normalized plots [17] for the pseudorapidity of charged particles for $N_{\text {ch}} \ge 1$ (upper left), and $N_{\text {ch}} \ge 6$ (lower left), for charged particles with $p_{\mathrm {T}} >500\,\text {Me}\text {V}$. The figure on the upper right shows a similar distribution for $N_{\text {ch}} \ge 2$, and the lower right for $N_{\text {ch}} \ge 20$, where the charged particles have $p_{\mathrm {T}} >100\,\text {Me}\text {V}$. ATLAS MB data are compared with the predictions from ${\textsc {herwig}} \,7$, with the $\text {SoftTune}$ and CH tunes. The coloured band in the ratio plot represents the total experimental uncertainty in the data. The vertical bars on the points for the different predictions represent the statistical uncertainties

**Fig. 22:**
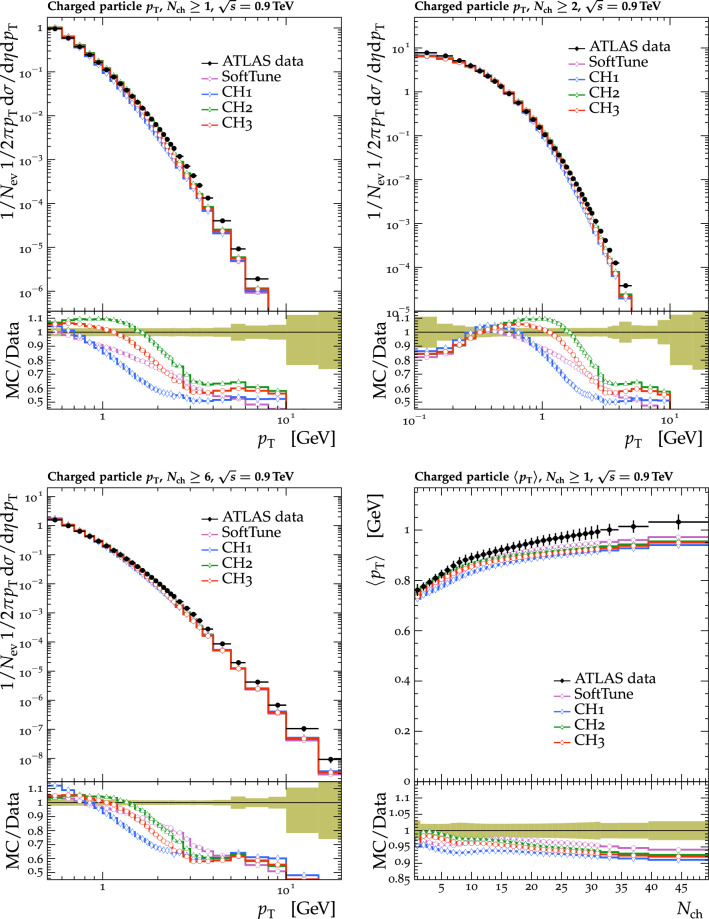
Normalized plots [17] for the charged-particle $p_{\mathrm {T}}$ for $N_{\text {ch}} \ge 1$ (upper left), $N_{\text {ch}} \ge 2$ (upper right), and $N_{\text {ch}} \ge 6$ (lower left). The mean charged-particle $p_{\mathrm {T}}$ as a function of the charged-particle multiplicity is also shown (lower right). ATLAS MB data are compared with the predictions from ${\textsc {herwig}} \,7$, with the $\text {SoftTune}$ and CH tunes. The coloured band in the ratio plot represents the total experimental uncertainty in the data. The vertical bars on the points for the different predictions represent the statistical uncertainties

**Fig. 23:**
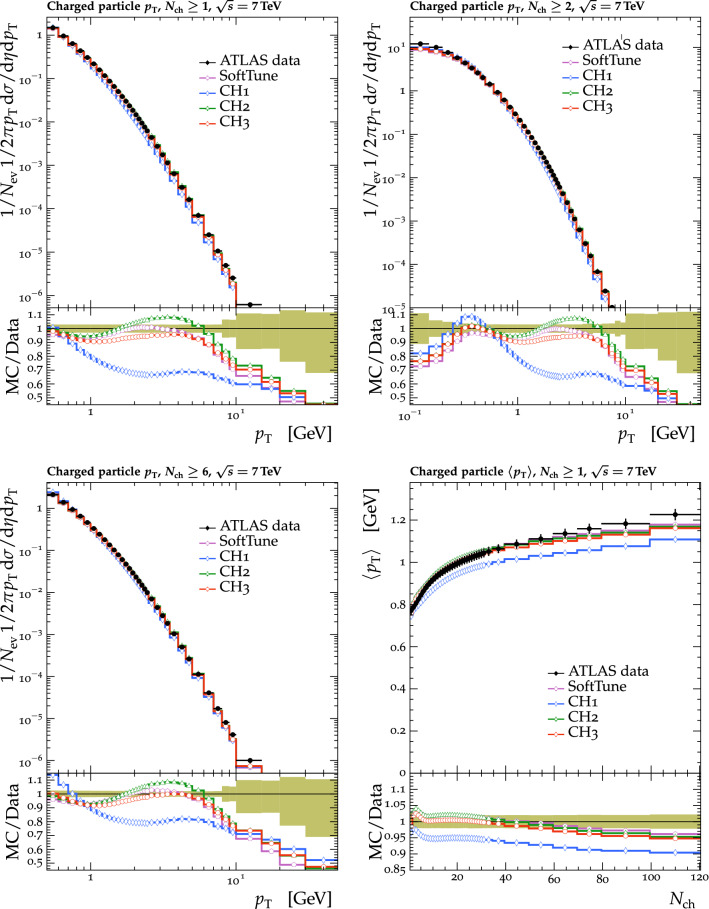
Normalized plots [17] for the charged-particle $p_{\mathrm {T}}$ for $N_{\text {ch}} \ge 1$ (upper left), $N_{\text {ch}} \ge 2$ (upper right), and $N_{\text {ch}} \ge 6$ (lower left). The mean charged-particle $p_{\mathrm {T}}$ as a function of the charged-particle multiplicity is also shown (lower right). ATLAS MB data are compared with the predictions from ${\textsc {herwig}} \,7$, with the $\text {SoftTune}$ and CH tunes. The coloured band in the ratio plot represents the total experimental uncertainty in the data. The vertical bars on the points for the different predictions represent the statistical uncertainties

**Fig. 24:**
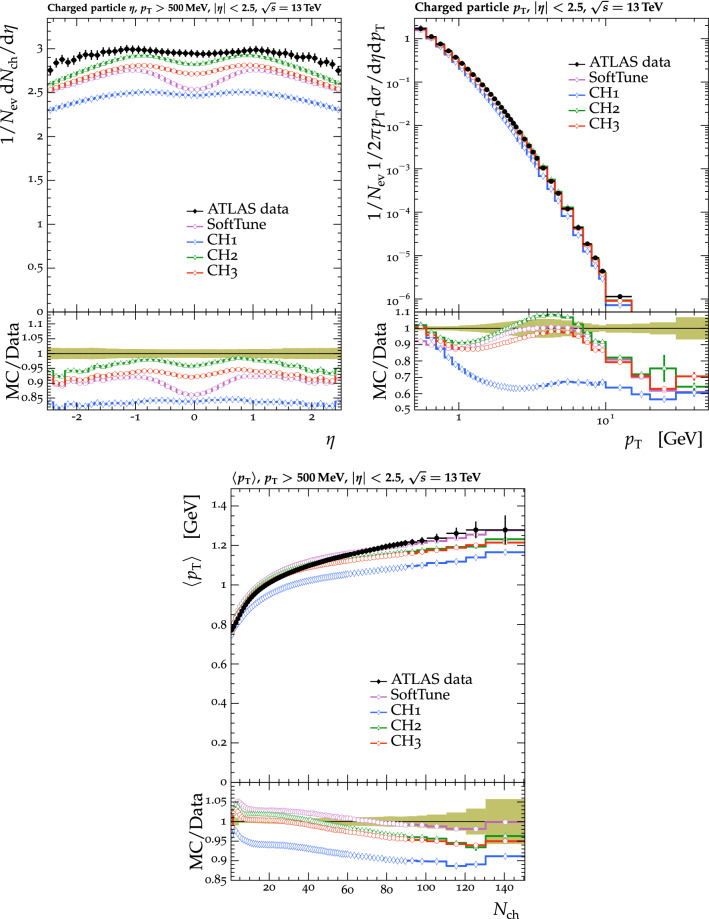
Normalized plots [48] for the pseudorapidity of charged particles (upper left), charged-particle $p_{\mathrm {T}}$ distribution (upper left), and the mean charged-particle $p_{\mathrm {T}}$ distribution as a function of the charged-particle multiplicity (lower), all for $|\eta |<2.5$. ATLAS MB data are compared with the predictions from ${\textsc {herwig}} \,7$, with the $\text {SoftTune}$ and CH tunes. The coloured band in the ratio plot represents the total experimental uncertainty in the data. The vertical bars on the points for the different predictions represent the statistical uncertainties

**Fig. 25:**
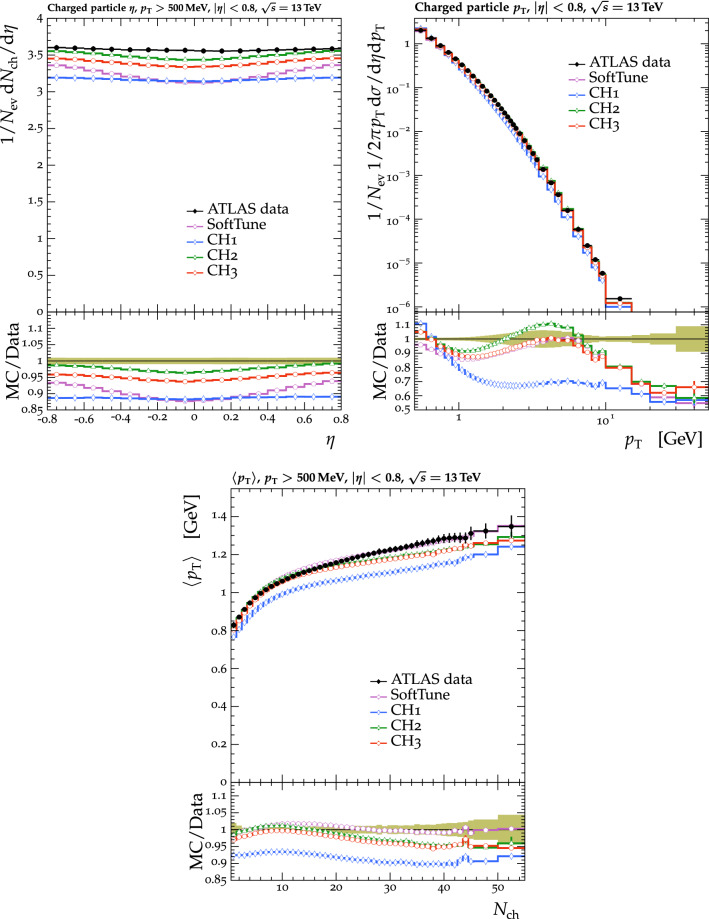
Normalized plots [48] for the pseudorapidity of charged particles (upper left), charged-particle $p_{\mathrm {T}}$ distribution (upper left), and the mean charged-particle $p_{\mathrm {T}}$ distribution as a function of the charged-particle multiplicity (lower), all for $|\eta |<0.8$. ATLAS MB data are compared with the predictions from ${\textsc {herwig}} \,7$, with the $\text {SoftTune}$ and CH tunes. The coloured band in the ratio plot represents the total experimental uncertainty in the data. The vertical bars on the points for the different predictions represent the statistical uncertainties

**Fig. 26:**
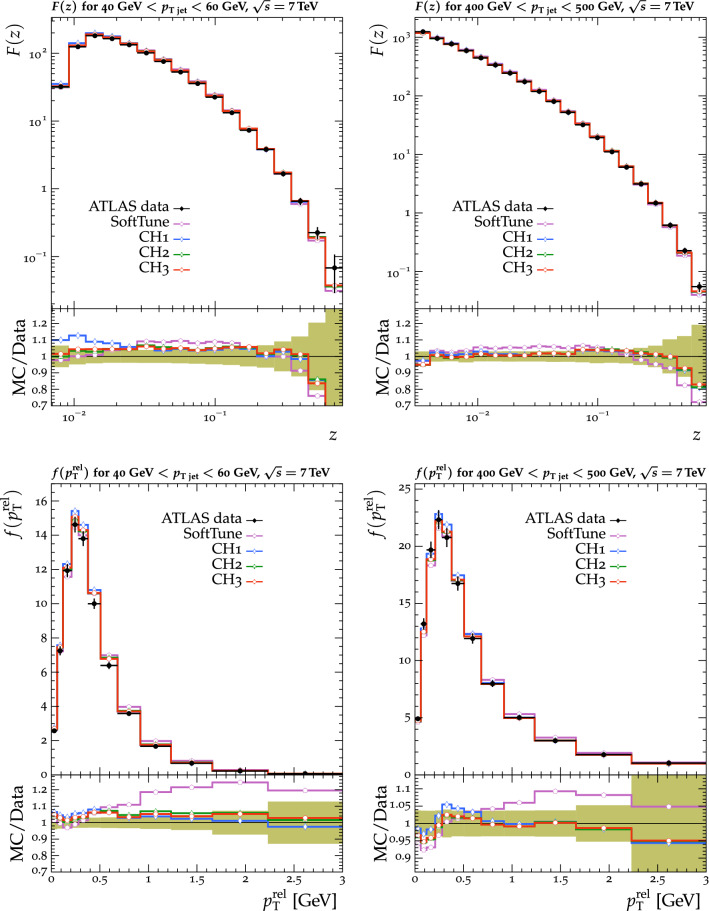
The ATLAS data at $\sqrt{s} =7\,\text {Te}\text {V}$ on the $F(z)$ and $f(p_{\mathrm {T}} ^{\text {rel}}$) distributions [17]. ATLAS inclusive jet data are compared with the predictions from ${\textsc {herwig}} \,7$, with the $\text {SoftTune}$ and CH tunes. The coloured band in the ratio plot represents the total experimental uncertainty in the data. The vertical bars on the points for the different predictions represent the statistical uncertainties

## Data Availability

This manuscript has no associated data or the data will not be deposited. [Authors’ comment: Release and preservation of data used by the CMS Collaboration as the basis for publications is guided by the CMS policy as written in its document “CMS data preservation, re-use and open access policy” (https://cms-docdb.cern.ch/cgibin/PublicDocDB/RetrieveFile?docid=6032&filename=CMSDataPolicyV1.2.pdf&version=2).]

## Appendix A: Comparison with ATLAS MB data

Figures 20, 21, 22, 23, 24 and 25 show comparisons of the tune predictions with MB data collected by the ATLAS experiment at $\sqrt{s} =0.9,~7,$ and $13\,\text {Te}\text {V}$, which were used in deriving the parameters of $\text {SoftTune}$. Figures 20 and 21 show the pseudorapidity distributions of charged particles at $\sqrt{s} =0.9$ and $7\,\text {Te}\text {V}$ respectively, for various minimum $N_{\text {ch}}$. Figures 22 and 23 show the charged-particle $p_{\mathrm {T}}$ distributions at $\sqrt{s} =0.9$ and $7\,\text {Te}\text {V}$ respectively, for various minimum $N_{\text {ch}}$. The distributions of mean charged-particle $p_{\mathrm {T}}$ as a function of the charged-particle multiplicity are also shown in Figs. 22 and 23. Figures 24 and 25 show the pseudorapidity and charged-particle $p_{\mathrm {T}}$ distributions at $\sqrt{s} =13\,\text {Te}\text {V}$, for $|\eta |<2.5$ and $|\eta |<0.8$ respectively. The corresponding distributions of the mean charged-particle $p_{\mathrm {T}}$ as a function of the charged-particle multiplicity are also shown in Figs. 24 and 25.

## Appendix B: Comparison with ATLAS inclusive jet events

Figure 26 shows the $F(z)$ distribution as a function of $z$, and the $f(p_{\mathrm {T}} ^{\text {rel}}$) distribution as a function of $p_{\mathrm {T}} ^{\text {rel}}$, as measured by the ATLAS experiment, along with the ${\textsc {herwig}} \,7$ predictions. The former distribution is a differential measurement of the charged-particle multiplicity inside jets as a function of the fraction of the jet longitudinal momentum carried by the jet constituents, $z$. The latter distribution is the same data but as a function of the transverse momentum of the jet constituents, $p_{\mathrm {T}} ^{\text {rel}}$, with respect to the jet axis. The jets are clustered using the anti-$k_{\mathrm {T}}$ algorithm with a distance parameter of 0.6, and the distributions are shown for two ranges of jet $p_{\mathrm {T}}$ ($p_{\mathrm {T}} ^{\text {jet}}$): $40< p_{\mathrm {T}} ^{\text {jet}} < 60 \,\text {Ge}\text {V}$ and $400< p_{\mathrm {T}} ^{\text {jet}} < 500 \,\text {Ge}\text {V}$. For all distributions, $\text {SoftTune}$ provides the least consistent prediction of the data. At low jet $p_{\mathrm {T}}$, the CH2 and CH3 tunes provide the best description of the data, whereas the CH1 tune deviates somewhat from the data both at low $z$ and at low $f(p_{\mathrm {T}} ^{\text {rel}}$). At high jet $p_{\mathrm {T}}$, only $\text {SoftTune}$ shows significant differences with respect to the data; however, these differences are smaller than those observed at low jet $p_{\mathrm {T}}$.
